# Comparisons of management practices and farm design on Australian commercial layer and meat chicken farms: Cage, barn and free range

**DOI:** 10.1371/journal.pone.0188505

**Published:** 2017-11-22

**Authors:** Angela Bullanday Scott, Mini Singh, Jenny-Ann Toribio, Marta Hernandez-Jover, Belinda Barnes, Kathryn Glass, Barbara Moloney, Amanda Lee, Peter Groves

**Affiliations:** 1 Sydney School of Veterinary Science, Faculty of Science, University of Sydney, Sydney, Australia; 2 School of Animal and Veterinary Science, Charles Sturt University, Sydney, Australia; 3 Quantitative Sciences, Department of Agriculture, Canberra, Australia; 4 College of Medicine, Biology and Environment, Australian National University, Canberra, Australia; 5 NSW Department of Primary Industries, Sydney, Australia; Animal and Plant Health Agency, UNITED KINGDOM

## Abstract

There are few published studies describing the unique management practices, farm design and housing characteristics of commercial meat chicken and layer farms in Australia. In particular, there has been a large expansion of free range poultry production in Australia in recent years, but limited information about this enterprise exists. This study aimed to describe features of Australian commercial chicken farms, with particular interest in free range farms, by conducting on-farm interviews of 25 free range layer farms, nine cage layer farms, nine barn layer farms, six free range meat chicken farms and 15 barn meat chicken farms in the Sydney basin bioregion and South East Queensland. Comparisons between the different enterprises (cage, barn and free range) were explored, including stocking densities, depopulation procedures, environmental control methods and sources of information for farmers. Additional information collected for free range farms include range size, range characteristics and range access. The median number of chickens per shed was greatest in free range meat chicken farms (31,058), followed by barn meat chicken (20,817), free range layer (10,713), barn layer (9,300) and cage layer farms (9,000). Sheds had cooling pads and tunnel ventilation in just over half of both barn and free range meat chicken farms (53%, n = 8) and was least common in free range layer farms (16%, n = 4). Range access in free range meat chicken farms was from sunrise to dark in the majority (93%, n = 14) of free range meat chicken farms. Over half of free range layer farms (56%, n = 14) granted range access at a set time each morning; most commonly between 9:00 to 10.00am (86%, n = 12), and chickens were placed back inside sheds when it was dusk.

## Introduction

Aspects of the Australian commercial poultry industry, including management practices, housing characteristics and range features are similar to poultry systems in other developed countries and others are distinctively different. Of note is the significant expansion of free range poultry production of both meat chicken and eggs in Australia in recent years. This expansion is largely due to increased demand by the Australian public, where it is perceived that products produced in less intensive systems are of higher welfare status compared to products produced in intensive systems [1].

Since 2012, the Australian retail turnover of free range eggs has surpassed that of cage, barn and organic eggs; with the latest percentages recorded in 2015 at 49%, 39%, 9% and 3%, respectively. However, cage eggs still surpass free range eggs in terms of volume of eggs produced; at 52% cage eggs compared to 39% free range [2]. The European Union (EU) implemented a ban on battery cages in 2012, and farms must convert to enriched cages or use alternative systems such as barn or free range [3]. In the United Kingdom (UK) this has lead a significant increase in free range poultry production. The volume of eggs produced by cage farms in the UK surpasses free range farms to a similar degree to Australia; at 52% and 44% in 2015 respectively [4]. In the United States of America (USA), the majority of laying hens (approximately 95%) are housed in conventional cage systems. However, there are current shifts within individual states to implement cage bans [5].

Australian free range meat chicken production has also grown to at least 15% of the total market in 2015 from being regarded as a ‘cottage industry’ in 2006 [6]. This growth is significant compared to other developed countries. In the UK, free range chicken meat production appears to be on a gradual decline. The country’s total meat chickens reared on free range systems was 6%, 5% and 3% in 2001, 2013 and 2014 respectively [7–9]. In the USA, less than 1% of the country’s total meat chickens were free range in 2012. However organic chicken farms are also recognized as free range and the organic food market has experienced strong growth since the establishment of the National Organic Program in 2002. In addition, organic chicken was recognized as the leading organic meat in terms of growth and so the total flock percentage identified as free range may increase in the future [10, 11]. In Australia, there is a high degree of private ownership within the Australian egg industry, leading to great variation between farms. In contrast, the Australian meat chicken industry is vertically integrated. Two large companies supply more than 70% of Australia’s meat chicken, and they have created their own standards in conjunction with third-party certifiers [12]. The consumer-driven expansion of free range production in Australia has led to the conversion of older, conventional farms into free range systems, as well as the development of new free range farms. Details on management practices and farm design across Australian commercial chicken farms, particularly free range enterprises, is lacking in the literature. It is particularly important to capture this information given the significant expansion of free range enterprises; this will help inform decisions related to the management of animal health.

This study reports the variations in management practices, farm design and housing characteristics across commercially operated Australian layer and meat chicken farms that were captured during on-farm visits. Comparisons between the different enterprises (cage, barn and free range) are explored and unique features of free range farms are described.

## Methods

A survey was conducted on commercial chicken farms in the Sydney basin and South East Queensland regions of Australia from June 2015 to February 2016. Commercial layer farms were defined as those having more than 1,000 birds, and commercial meat chicken farms were defined as those having more than 25,000 birds. The survey involved farm visits by the researchers and face-to-face interviews and observations. The experimental procedures used for this study were approved by the Human Ethics Committee of the University of Sydney, Australia and all results obtained were kept confidential (ethics reference number: 2015/252).

### Region and farm selection

The survey was purposely conducted in the state of New South Wales as it has the highest levels of layer and meat chicken production in Australia. As of December 2014, approximately 120 (48%) of the 252 commercial layer farms in Australia were located in NSW, and NSW was the leading state in terms of volume of meat chicken produced (34%) [13, 14].

Within NSW, the Sydney basin bioregion was selected for the survey. Bioregions are large land areas defined by natural features and environmental processes. From north to south, the Sydney basin bioregion extends from just north of Newcastle (latitude approximately -32.4367) to just north of Bateman’s Bay (latitude approximately -35.6132) and almost as far west as Mudgee (average longitude approximately 150.1765) [15]. The Sydney basin bioregion was chosen due to its high density and variety of layer and meat chicken farms and its history of an Avian Influenza (AI) outbreak in Maitland in 2011 [16]. It was later found that numbers of free range meat chicken farms were limited in this region and were all owned by one of the two large Australian meat chicken companies. Therefore permission was sought to visit free range meat chicken farms owned by the other large Australian meat chicken company in the state of Queensland.

A comprehensive list of commercial layer and meat chicken farms and their contact information in these regions was created by compiling farm lists from various sources. Permission was sought from corporations, consultants, veterinarians and companies to provide such lists and sharing of this information was refused on only one occasion. It was considered that the final compiled list included the vast majority of layer and meat chicken farms in the Sydney basin bioregion and all free range meat chicken farms owned by the one company in the South East Queensland region. Due to logistic and budgetary constraints, the total number of farms that could be surveyed was 80 farms. As information in the literature on free range farms is lacking compared to other types of farms, a higher proportion of free range farms were included. In addition, information in the literature describing variation between layer farms that is possible with the high level of private ownership within the Australian egg industry is also lacking, hence supporting the decision of including a high proportion of layer farms. The target number set for each farm type was 25 free range layer farms, 10 cage layer farms, 10 barn layer farms, 15 free range meat chicken farms and 15 barn meat chicken farms.

Farms from the final compiled list were sorted by farm type. Random selection of farms was then conducted within each farm type. Selected farms were contacted via telephone, the project explained to them, and their consent to participate in the project was requested. If the request was rejected, the next farm on the randomly selected list was contacted. If the request was accepted, a date and time was arranged for the farm visit.

### Questionnaire development

Two questionnaires were created: a main questionnaire and a biosecurity questionnaire, both written in English. The main questionnaire consisted of six sections: farm information, water, poultry health, range, wild birds and other wild animals, with a total of 102 questions. It comprised short closed, semi-closed and open questions, and was completed during a face-to-face interview estimated to take one hour. The biosecurity questionnaire consisted of 2 sections: communication, and biosecurity practices performed on the farm. The communication component consisted of questions related to where farmers source information from regarding poultry health and industry news and also how they receive that information. Examples of sources of information include poultry organisations, integrators and veterinarians. Examples of information delivery include newsletters, emails, social media and television/radio. The biosecurity questionnaire overall consisted of seven closed questions, including personal ratings of the importance of specified biosecurity practices, and was self-completed by the farmer in approximately 15 minutes. The questionnaire was pilot tested on two local farms; a free range layer and cage layer farm, to evaluate clarity and wording of the questions which were then modified appropriately. The data from these pilot tested farms was included in the survey. Copies of the questionnaires are available in the supporting information (S1 Text & S2 Text).

### Farm surveys

Each farm survey took one to two hours depending on the size and type of the farm. The farm survey consisted of a face-to-face interview conducted by the researchers using the main questionnaire, followed by the farmer filling out the biosecurity questionnaire with assistance available from the researchers if required. After the interviews, researchers recorded visual observations including shed design, range design, topography, waterbody locations and biosecurity practices. Vegetative cover on the range areas was estimated by the researchers as an estimated percentage of the total range area based on observation during the visit. The stocking density for cage layer farms was calculated by dividing the number of chickens per shed by the shed area covered by cages multiplied by the number of tiers. This was done to avoid the falsely high stocking density on multi-tier cage farms which would be obtained when simply dividing the number of birds with the shed area, as done on all other farm types. For measurements of distances, such as between sheds, these were estimated using Google Maps (2016 Google Inc., California, USA) after the farm visit.

### Data analysis

After each farm visit, data from the questionnaires and observation records were entered in Microsoft Access (Microsoft, PC/Windows 7, 2010, Redmond, WA, USA) and checked for data entry errors. The statistical program JMP was used (SAS Institute Inc., 2010, Cary, USA) for all statistical analyses of the data. One-way analyses of variance were used to determine any significant statistical differences in farm design factors between the different farm types. P-values were used to detect any statistical significance between different factors and a p-value of <0.05 was considered significant.

## Results

### Farm size

The farms surveyed were all commercially operating farms. The largest farm visited was a cage layer farm with 467,000 birds and the smallest was a free range layer farm with 1,450 birds. The median number of chickens was greatest on meat chicken farms (140,600 and 88,000 chickens for free range and barn meat chicken farms respectively). This was then followed by cage layer, free range layer and barn layer farms (40,000, 32,000 and 17,500 chickens respectively). All meat chicken farms had at least two commercial poultry sheds on the farm whilst all layer farms had at least one. Meat chicken farms had a greater median number of sheds on the farm compared to layer farms as shown in Table 1.

**Table 1:** The median and range for the numbers of chicken and shed attributes for barn and free range meat chicken farms, and for cage, barn and free range layer farms in the Sydney basin bioregion and South East Queensland, Australia, June 2015 until February 2016.

Farm attribute			Farm type		
	Barn meat chicken (n = 15)	Free range meat chicken (n = 15)	Cage layer (n = 9)	Barn layer (n = 9)	Free range layer (n = 25)
Number of sheds on farm surveyed	4 (2–10)	5 (2–12)	2 (1–12)	2 (1–10)	3 (1–16)
Total number of chickens on farm surveyed	88,000 (29,000–210,000)	140,560 (67,000–271,000)	40,000 (7,500–467,000)	17,500 (5,000–90,000)	32,000 (1,450–163,000)
Number of chickens per shed	20,817 (14,450–32,067)	31,058 (13,400–50,280)	9,000 (3,986–71,000)	9,300 (5,000–17,500)	10,713 (250–18,000)
Shed area (m^2^)	1,453 (870–3,142)	1,937 (876–3,145)	1,500 (995–2,500)	1,064 (720–1,750)	1,192 (15^a^-1,820)
Stocking density inside shed (birds/m^2^)	13.6 (8.1–20.5)	16.0 (15.3–18.6)	8.7 (3.8–11.4)	9.0 (6.7–11.0)	10.2 (1.2–33.3^a^)

^**a**^ The farm with this shed area and stocking density had mobile caravan type sheds. Details are under ‘shed area and design’.

### Shed information

#### Shed area and design

The median shed area was largest in free range meat chicken farms (1,937m^2^) and smallest in barn layer farms (1,064 m^2^). The smallest sheds across all farm types was 15 m^2^ in the form of mobile caravans on one free range layer farm. These are roadworthy and are moved on to different areas of pasture when pasture is denuded. One mobile caravan type farm was visited in this study. Features of mobile caravan type farms involve limited space inside the caravans, but they are kept open for most of the day. In addition, nest boxes are provided inside but food and water is only available outside of the shed, thereby driving chickens to go outside.

Variation in shed design was greatest amongst the layer farms. Some layer sheds were divided internally into sections by wire mesh. In this design, groups of chickens were separated physically in these sections but could still see and interact, to a limited extent, with other groups. These shed designs were only found in 33% (n = 3) and 16% (n = 4) of barn and free range layer farms respectively.

Features of shed flooring were similar amongst the meat chicken farms. All meat chicken farms used litter on the floor of the sheds, most commonly wood shavings (71%), followed by saw dust (23%) and rice hulls (6%). In contrast, all barn and free range layer farms used slat type flooring which covered 100% of the flooring in the house for the chickens. The majority of slats were made out of plastic (94%), followed by wire (6%). The housing of all cage layer farms used solid flooring.

The median number of tiers or levels on cage farms was three, and this ranged from one to seven. The median number of rows was six (4–15). All cage rows were ‘back to back’ i.e. each row had an adjacent row attached before a space in between the rows. The median number of cages per row, excluding the adjacent row, was 122 (70–164). There was a median of 2,250 cages per shed (840–11,808). The median number of chickens per cage was four (3–7).

#### Capacity and stocking density

The median number of chickens per shed was greatest in free range meat chicken farms (31,058), followed by barn meat chicken (20,817), free range layer (10,713), barn layer (9,300) and cage layer farms (9,000). The number of chickens per shed ranged up to 71,000 chickens, which was found on a cage layer farm. The smallest capacity sheds were found on a free range layer farm, housing approximately 250 chickens (Table 1).

The median stocking density was greatest in meat chicken farms (13.6 and 16.0 birds/m^2^ for barn and free range meat chicken farms respectively) compared to layer farms, where it was smallest in cage layer farms (8.7 birds/m^2^) (Table 1). A high stocking density of 33.3 birds/m^2^ was found on the one mobile caravan farm. As mentioned previously, space is limited inside the caravans. Perches are placed on multiple levels inside, thereby providing hens with more roost areas. The caravans are only in full use at night time when the doors are closed for shelter and protection.

#### Drinkers, feeders and nests

Nipple type drinkers were used on 97% of farms. They were present on all barn meat chicken, cage layer and barn layer farms. One free range meat chicken farm used a combination of nipples and cup type drinkers and one free range layer farm used large bell type drinkers. Taking all drinker types into account, there was a median of 9 chickens per drinker overall for all farm types. There was no statistically significant difference in the number of chickens per drinker between the different farm types (P = 0.2).

Automatic pan type feeders were used on all meat chicken farms. On barn layer farms, automatic pans were used on 56% (n = 5) and chain feeders were used on 44% (n = 4) of farms. Travelling hoppers were the most common feeder type on cage layer farms (44%, n = 4), followed by manual troughs (33%, n = 3). One cage layer farm used a combination of chain feeders and travelling hoppers, and another used manual troughs with travelling hoppers. Chain feeders were most commonly used on free range layer farms (52%, n = 13), followed by automatic pans (36%, n = 9), gravity pans, (8%, n = 2) and then a combination of automatic pans with chain feeders (4%, n = 1). There was a median of 54 chickens per automatic pan and 4 cm of chain feeder per bird overall. There was no statistically significant difference in the number of automatic pans and length of chain feeder per chicken between the different farm types (P = 0.07 and P = 0.44 respectively).

Eggs were collected by conveyer belt on 90% of layer farms. Manual egg collection was performed on 10% of layer farms, all of which were cage layer farms. There was a median of 79 chickens per nest across barn and free range layer farms. There was no statistically significant difference in the number of chickens per nest between the different farm types (P = 0.2).

#### Environmental control methods

All meat chicken farms had heaters in the shed that were mainly used for heating brooding areas for new chicks. No layer farms had heaters. A variety of environmental control methods to cool down birds during hot weather was reported across all farm types. Cooling pads and tunnel ventilation were reported in just over half of both barn and free range meat chicken farms (53%, n = 8) and was least common in free range layer farms (16%, n = 4). Both foggers and stirring fans were reported in over half of every farm type. Curtains and/or shutters used to block or allow outside breeze was found in over half of all farm types with the exception of cage layer farms where these were present in only 22% (n = 2). These values are shown in Table 2.

**Table 2:** A description of the environmental control methods used by cage, barn and free range meat chicken and layer farms inside sheds, in the Sydney basin bioregion and South East Queensland, Australia, June 2015—February 2016.

Environmental control method	Farm type				
	Barn meat chicken (n = 15)	Free range meat chicken (n = 15)	Cage layer (n = 9)	Barn layer (n = 9)	Free range layer (n = 25)
Cooling pads/ tunnel ventilation (% farms)	53 (n = 8)	53 (n = 8)	33 (n = 3)	33 (n = 3)	16 (n = 4)
Foggers (% farms)	60 (n = 9)	80 (n = 12)	67 (n = 6)	78 (n = 7)	84 (n = 21)
Curtains/ shutters (% farms)	67 (n = 10)	100	22 (n = 2)	78 (n = 7)	84 (n = 21)
Sprinklers on roof (% farms)	7 (n = 1)	0	56 (n = 5)	11 (n = 1)	44 (n = 11)
Stirring fans(% farms)	93 (n = 14)	100	56 (n = 5)	100	88 (n = 22)

### Chicken management and health

#### Age groups

Only one age group of birds per shed was present in all meat chicken farms. There was a median of one age group per shed for both barn layer and free range layer farm types, and a maximum of seven and four age groups respectively. Multiple ages in one shed in these farm types were in sheds divided internally by wire mesh as described previously. Each section was a different age group but each group could see and interact through the wire mesh. It was found that 78% (n = 7) of cage layer farms had multiple age groups in one shed, with a median number of four age groups per shed.

The median number of age groups per farm was one for all meat chicken farms. The maximum number of age groups was ten on one barn meat chicken farm, due to the large size of the farm. For cage, barn and free range layer farms, the median number of age groups per farm was five, three and four respectively.

#### Chicken breed and rearing

Meat chicken farms most commonly used a combination of the Ross and Cobb chicken breed on the one farm (53%, n = 16), followed by exclusive use of the Cobb breed (27%, n = 8) and then the Ross breed (20%, n = 6). The ISA Brown breed was the most common breed on the layer farms (77%, n = 33) and was the most common breed per layer farm type. Other layer breeds used were the Hyline (30%, n = 13), Lohmann (7%, n = 3), Leghorn (5%, n = 2), Hisex (2%, n = 1) and Heritage breeds (2%, n = 1), and some farms used a combination of different breeds on the one farm. A minority (14%, n = 7) of layer farms had a rearing shed on the farm in which they rear their own birds. Therefore most layer farms relied on outside rearing farms for production layers. All meat chicken farms received new birds at one day old from the hatcheries.

#### Health records and vaccinations

All meat chicken farms kept written health records documenting aspects such as the number of mortalities per day. Written health records were kept on 89% (n = 8), 67% (n = 6) and 96% (n = 24) of layer barn, cage and free range farms respectively.

All farms vaccinated chickens against Newcastle disease virus and Infectious Bronchitis virus. Half (50%, n = 15) of meat chicken farms vaccinate against Marek’s disease (MD). Infectious laryngotracheitis (ILT) was only vaccinated when needed in meat chicken farms and had been performed in 17% (n = 5) of farms at the time of the visits. In contrast, all layer farms were vaccinated routinely against ILT, as well as Egg drop syndrome (EDS). Different vaccination protocols existed across the layer farms dependent on factors such as the company of the farm, the source of chickens and/or the veterinarian associated with the farm. Vaccinations are also performed at various stages of the commercial layer lifetime, including during the rearing stage. Vaccination percentages in layer farms against the following diseases were: IBD 24% (n = 24), Fowl pox 93% (n = 39), *Mycoplasma gallisepticum* 62% (n = 26), *Mycoplasma synoviae* 40% (n = 17), MD 83% (n = 35), coccidiosis 12% (n = 5), infectious coryza 26% (n = 11), avian encephalomyelitis 93% (n = 39) and *Salmonella* Typhimurium 14% (n = 6).

#### Veterinarian contact

Veterinarian visits for advice on farm improvements were described to occur at least annually and occurred in addition to disease investigation. These were reported to occur in more than half (57%, n = 17) of meat chicken farms and in less than half (42%) of layer farms. Other farms reported that they contact the veterinarian only on the occasion of a health issue or disease investigation.

#### Depopulation, dead bird and manure management

A thinning out procedure occurs on 90% (n = 27) of meat chicken farms surveyed. The process of thinning out generally involves a third of birds being removed at around 30 days of age, with a second pick up a week after and a final third pick up a week after that. However, this varied considerably depending on what orders are made further in the production chain. Some birds were picked up as early as 21 days for spatchcock, or small bird, orders and some birds were left as late as 55 days for large piece orders. Ninety-two percent (n = 23) of free range and 89% (n = 8) of both, cage and barn layer farms removed all chickens of the same age group on one day during depopulation. In the case of multiage cage layer farms, each row or tier was one age group and so only that age group was removed during depopulation. In the case of barn and free range layer farms with sheds that have multiple age groups in one shed separated by wire mesh, one age group i.e. one section of the shed, would be depopulated at the appropriate times. Of those farms that do not depopulate a whole age group at one time, it either occurred over two to three days because of the volume of birds, birds are kept until they die naturally or were sold individually to different customers for meat.

Dead birds were most commonly placed in dead bird freezers across all farm types (44%, n = 32). Dead bird collectors emptied these freezers once full. Freezing dead birds was followed by composting dead birds on or nearby the farm (32%, n = 23) and burial on farm (12%, n = 9). Less common answers included incineration, placing dead birds in bio-bins and sending dead birds to the tip. Some farms performed a combination of methods. Manure was most commonly removed and given to an off-site user across all chicken farms (86%, n = 63). Less common answers included composting on farm, stockpiling on farm and spreading on paddocks.

### Sources and delivery of information to farmers on poultry industry and poultry health

For the communication section of the biosecurity questionnaires, although researchers were present to discuss and explain the questionnaire to the farmers, some farmers chose to leave some questions blank even after discussion. Of those that answered, most farmers (73%, n = 46/63) across all farm types stated that they communicate with other poultry farmers for information regarding poultry health and industry news. Overall, farmers rated other poultry farmers as a ‘moderately reliable’ source of information.

The majority (86%, n = 50/58) of farmers used poultry organisations for news about the poultry industry and this received an average rating of ‘very reliable’. Organisations used include the NSW Farmers Association, the Australian Chicken Meat Federation, Australian Egg Corporation Limited (AECL), NSW Food Authority, World Poultry Science Association and the Poultry Cooperative Research Centre. Poultry integrators, or companies, were also a common source of information for farmers (96%, n = 52/54). Integrators also received an overall average rating of ‘very reliable’. The most reliable source of information rated by farmers which received an average rating of ‘extremely reliable’ was veterinarians; where 97% (63/65) reported to use them as a source of information. Newsletters were a common source of information delivery, used by 87% (n = 40/46) of farmers. Less common answers include subscribed emails, social media, television/radio and websites. Newsletters include Poultry Digest, AECL, NSW food authority and in-house company magazines.

### Farm services and contractors

For meat chicken farms, the delivery of day old chicks was usually performed by the company’s own specialized trucks. One private contractor was also identified. For layer farms, one private contractor delivered reared birds to all layer farms except for two layer farms which had a different private contractor each.

Depopulation on meat chicken farms was performed by private contractors that differed depending on the meat chicken integrator. Integrators were found to have up to three private contractors. Of these, a private contractor specializing in the depopulation of spent layer hens was used in 86% (n = 37) of layer farms.

There were usually private contractors for the delivery of fresh litter and shed sanitization for meat chicken farms. Shed sanitization was performed by on-site farm staff on 84% (n = 36) of layer farms. Across all farm types, electrical work was performed by off-site electricians (86%, n = 63) and in other cases the farmers were electricians themselves. Plumbing work was performed by off-site plumbers in over half (53%, n = 39) of poultry farms surveyed, where other poultry farmers commonly reported fixing plumbing issues themselves.

The removal of dead birds and manure was usually performed by private contractors for all farm types. There were a relatively large number of different contractors for manure and dead bird removal across all farm types. These contractors tended to work in small local areas, where it was common to only collect from one or two farms.

### Free range information

#### Style and range size

All commercial free range meat chicken farms surveyed had a fixed range dedicated for each shed of birds. All range areas were rectangular in shape, adjacent and aligned with the length of the shed. In comparison, over half (52%, n = 13) of free range layer farms rotate birds between ranges. In this case parts of the range are available for ranging and others are fenced off from the birds to allow the pasture to recover. Rotation of ranges usually occurs between new batches of birds and this is performed to allow recovery of vegetation on used range areas. The shape of the ranges varied considerably among the free range layer farms. All free range layer sheds had dedicated range areas per shed. The median range area for free range meat chicken farms (2,476.5m^2^) was smaller than that of free range layer farms (18,000m^2^) as shown in Table 3.

**Table 3:** The median and range number of stocking density on the range area and range-related attributes for free range meat chicken and layer farms in the Sydney basin bioregion and South East Queensland, Australia, June 2015 until February 2016.

Range attribute	Farm type	
	Free range meat chicken (n = 15)	Free range layer (n = 25)
Range area (m^2^)	2,476.5 (1170–5503.75)	18,000 (525–53,000)
Stocking density on range area (birds/m^2^)	12.8 (9.1–16)	0.7 (0.025–6.7)
Stocking density on range area (birds/ha)	127,692 (91,355–159,972)	6590.9 (250–66,666.7)
Farmer reported proportion of birds in shed that use range (%)	40 (14.3–50)	50 (12.5–100)
Farmer reported proportion of range used by birds (%)	100 (50–100)	40 (10–100)
Shed wall covered by pop-holes (%)	50 (15–100)	60 (15–100)
Pop-holes per wall	7 (1–14)	5.5 (1–19)
Walls with pop-holes	1 (1–2)	1.5 (1–2)
Grass-cover on range (%)	80 (30–100)	60 (0–100)

#### Stocking density and range use

The median stocking density of free range meat chicken farms was 12.8 birds/m^2^ in comparison to free range layer farms at 0.7 birds/m^2^ (Table 3). Farmers reported a greater proportion of birds on free range layer farms (50%) that went outside to use the range as compared to free range meat chicken farms (40%). The median proportion of range used by the birds was 100% for free range meat chicken farms and only 40% for free range layer farms. This appears to relate to the smaller range areas found on free range meat chicken farms compared to free range layer farms (Table 3). These results are at one point in time during range access and does not account for individual birds entering and leaving the range continuously.

#### Pop-holes

Pop-holes, which are the openings on shed walls that allow chickens to access the range, varied in size and shape amongst the free range farms. The median proportion of the shed wall covered by pop-holes in meat chicken and layer free range farms was 50% and 60% respectively. Among the free range farms, three meat chicken and two layer farms had one entire wall of the shed that could be opened up to allow range access to the birds. The median number of pop-holes per wall on free range meat chicken farms (7) exceeded that of free range layer farms (5.5). Pop-holes were present on either one or two sides of the length of the shed for both farm types. The median number of walls covered by pop-holes for meat chicken and layer free range farms was 1 and 1.5 respectively (Table 3).

#### Range access

The majority (93%, n = 14) of free range meat chicken farms granted access for the birds to the range from sunrise to dusk. Total time allowed outside therefore varies between seasons where sundown times can vary from 5pm to 8pm in winter and summer, respectively [17]. Only one free range meat chicken farm opened pop-holes at a set time each morning (7:30am) and chickens were put back at dusk. Over half of free range layer farms (56%, n = 14) opened pop-holes at a set time each morning; most commonly between 9:00 to 10.00am (86%, n = 12) with one opening at 7.30am and another one at 10:30am. On all of these farms, chickens were placed back inside sheds when it was dusk. There were no specific times for bird range access on 28% (n = 7) of free range layer farms; farmers simply stated the birds were let out when it was light to dusk. Only four free range layer farms had specific times when birds were placed back inside, with this being between 5:30 to 8:00pm.

Access to the range for both farm types was restricted in certain weather conditions. Of the free range meat chicken farms, approximately half (53%, n = 8) did not allow birds outside when conditions were too cold, 87% when conditions were too hot and all of them when conditions were too wet. Wet conditions were defined as conditions in which pooling water was found on the range. For 40% (n = 6) of free range meat chicken farms, birds the temperature range which defined too cold or hot conditions was 17 to 28°C. Other farmers would allow birds outside depending on their own judgment about whether or not birds could tolerate the conditions. Ninety-two percent (n = 23) of free range layer farms did not allow birds outside during severe weather; the rest of farms reported chickens being allowed outside regardless of weather conditions. Severe weather included descriptions such as strong winds, thunderstorms and hail. While all free range layer farms allowed chickens out during cold conditions, two farms did not allow birds outside in hot conditions and 44% did not allow birds outside in wet conditions. Another factor to consider for birds being allowed outside is the age of the birds, with a median age at which meat chickens and layers being granted range access of 21 days and 22 weeks, respectively, limited to overall time birds have access to the range during their production cycle.

#### Waterbodies on range

All free range farms except one layer farm did not have waterbodies present on the range, with ranges being usually fenced off from waterbodies. Forty percent (n = 6) of free range meat chicken farms, the edge of the range was reported to be 50 to 250m from a waterbody. In the majority of free range layer farms (60%, n = 15), the edge of the range was <50m from a waterbody. Holes and drains on the range that fill up with water were reported by farmers in 47% (n = 7) and 96% (n = 24) of free range meat chicken and layer farms respectively.

#### Vegetation

Vegetative cover on the range was referred to as ‘high’ if the range had at least 50% of the area covered by grass or trees. Based on researcher observation on the day of visit, this was found on 60% of free range meat chicken farms (n = 9) and 68% of layer farms (n = 17). No vegetative cover was found on one free range layer farm. Range was bare next to the shed in 92% of free range layer farms (n = 23). The bareness usually extended to a distance that was similar to the width of the shed. Grass was uniformly distributed in 67% of free range meat chicken farms (n = 10). The median percentage of range covered by grass in free range meat chicken and layer farms was 80% and 60% respectively (Table 3).

Trees were scattered in 60% of free range layer farms (n = 15) and 87% of meat chicken farms (n = 13). Thirty-two percent of free range layer farms (n = 8) had trees surrounding the edge of the range only and no trees at all were seen on 13% free range meat chicken farms (n = 2).

#### Artificial structures and enrichment

Artificial shades covered a moderate (10–50%) amount of range area in 76% of free range layer (n = 19) and 80% meat chicken farms (n = 12). All other free range farms had either no or minimal (0–10%) artificial shade. Shade structures were most commonly flat, rectangular shades for both free range meat chicken (80%, n = 12) and layer farms (44%, n = 11). This was followed by arched shade structures for free range meat chicken farms (33%, n = 3). Flat shades built as an extension of the shed and arched shade structures were found in 20% (n = 5) and 16% (n = 4) of free range layer farms respectively. Artificial shade structures are located close to the sheds in the majority of free range layer farms (52%, n = 13), followed by being uniformly distributed throughout the range (36%, n = 9). Shade structures are uniformly distributed in the majority of free range meat chicken farms (67%, n = 10), followed by being close to the shed (20%, n = 3).

Deliberate placement of range enrichment items was provided in 40% (n = 6) of free range meat chicken farms and 20% (n = 5) of free range layer farms. Such items were usually hay bales and ladders.

#### Range management

Range cleaning and maintenance was reported on all free range meat chicken farms and involved mowing and slashing. In contrast, 20% (n = 5) of free range layer farms report maintenance of the range was not performed. Sixty percent (n = 9) of free range meat chicken farms reported that birds never escape the perimeter fencing of the range, while 33% (n = 5) reported this rarely occurs, and one farm reported it occurs often. Sixty eight percent (n = 17) of free range layer farms reported that chickens rarely escaped the perimeter fencing, while frequent escapes and no escapes were both reported in 16% (n = 4) of free range layer farms.

No free range meat chicken farms provide food or water on the range as compared to 12% (n = 3) of free range layer farms that did provide food or water on the range.

## Discussion

This paper improves knowledge of commercial chicken farms in Australia by documenting management practices, farm design and housing characteristics based on interviews and observation conducted on-farm, and including all types of farm enterprises in the layer sector and the chicken meat sector of the commercial chicken industry today. The sample of farms surveyed is likely representative of the commercial chicken egg and meat farms across Australia, given that NSW is the leading state in both the number of farms and volume of product produced for meat chicken and egg farms in Australia [14, 18, 19], and that the surveyed farms included a large range of farm sizes and a range of companies. However, farms located in the Sydney basin region are generally older with not as many tunnel ventilated sheds compared to farms located in other states in Australia. This must be considered in this study as most farms interviewed were located in the Sydney basin region.

For the layer industry in Australia, the total number of commercial farms has notably reduced in the last decade with approximately 600 farms recorded in 2007 compared to 252 farms at the time of writing; and some further reduction is expected. The on-farm interviews were conducted on layer farms ranging from small, independently operated farms to farms owned by the largest egg producing company in Australia, which produces 20% of the national market [20]. For meat chicken farms, permission for on-farm interviews was granted by three of seven Australian meat chicken processing companies, with two included being the largest companies in Australia that together supply more than 70% of Australia’s meat chicken [6].

This study focused on collecting information about commercially-operating farms as these produce the vast majority of chicken eggs and meat in Australia compared to backyard flocks. The estimated population of egg laying chickens in commercially operated farms in Australia is over 23 million and there is an estimated 512 million meat chickens slaughtered in Australia each year [6, 13]. The large majority of both chicken eggs and meat is purchased and consumed within Australia with minimal exports [12]. The last census of backyard poultry ownership occurred in 1992 in Australia and it was estimated that 7% of all households keep backyard poultry; with an estimated population of 1 million birds [12]. This figure is likely to be higher now with the increasing popularity of organic and free-range products [2, 6]. However, the majority of birds in backyard flocks are used to produce eggs for the owner’s personal consumption and the number of backyard chickens used for poultry meat at home is insignificantly small in Australia. Information on management practices and design of backyard poultry production is also lacking in the literature but is likely to vary greatly due to private ownership. There are concerns from the industry that the rise in backyard poultry production could lead to more disease introductions in commercial poultry farms. However, there is little contact between commercial poultry operations and backyard poultry production [12]. The national farm biosecurity manual for poultry production states that farm workers must not have any contact with avian species or pigs outside of the farm. If this occurs then workers must shower and change into new protective clothing prior to entering the farm [21].

In general, management practices and farm design were found to be more similar across the individual meat chicken farms compared to the layer farms in this study. This is considered to be due to the high level of private ownership amongst layer farms, the greater number of layer farm types, and the vertically integrated nature of the meat chicken industry. Companies in the meat chicken industry own or control most aspects of the supply and production chain, including genetic breeding stock, parent breeders, hatcheries, grow outs, processing plants and feed mills [6, 12]. This is comparable to the EU and USA, where the top ten companies together supply almost 30% and over 60% of the domestic markets respectively [22].

The median stocking density inside sheds for non-free range meat chicken and free range meat chicken was 13.6 and 16.0 birds/m^2^ respectively. One study assessed the performance of meat chickens at varying stocking densities in New Zealand; at 5, 10, 15 and 20 birds/m^2^. It was found that there was no linear relationship between stocking density and productivity per bird basis in terms of feed/weight gain, mortality or carcass characteristics [23], in accordance with other studies [24, 25]. There are however some welfare implications at high stocking densities, such as over 20 birds/ birds/m^2^, related to litter quality leading to poor gait and hock and foot pad burns [23].

All meat chicken farms had one age group per shed and 90% (n = 27) of farms performed thinning out compared to all-in-all-out during depopulation. Thinning out is performed to satisfy market demand for light and heavy meat chickens, improve space utilization and increase profits for the producer. However, risks associated with thinning out include biosecurity breaches, gut problems after fasting the whole shed followed by re-feeding remaining birds, and detrimental effects on meat quality. Biosecurity breaches can occur when bird-catcher crew members fail to follow biosecurity protocols when visiting multiple farms [12, 26, 27]. Nervousness in chickens leading to excess wing flapping can also occur after thinning out, which would lead to deep pectoral myopathy in the breast tissue can result, leading to a reduction in meat quality. There is a fine balance that must be achieved in order to optimize the benefits of thinning out procedures [26].

As well as concerns regarding biosecurity breaches for bird-catcher crew members, there are concerns for many other farm contacts. This study revealed there are a large number of different contacts for all farm types, including new bird deliverers, dead bird pick-up crews and tradesmen that visit other farms. Such contacts can introduce and spread poultry diseases between sheds and between farms, especially if biosecurity protocols are not followed. It is prudent all farms ensure visitors follow appropriate biosecurity practices to minimize the potential biosecurity breaches [12, 28].

There were multiple age groups in a shed on some layer farms; particularly those sheds that were divided internally by wire mesh and in cage layer farms. There are health concerns associated with multiple age groups per shed as certain diseases are able to transfer from old to new age groups. Similarly, pathogens can persist inside sheds if complete depopulation of sheds does not occur, where there is continuous replacement of old groups with new groups. Such pathogens include (MD) and avian influenza (AI). One of the highest concern in relation to AI is increased chance of mutating from low pathogenic AI to highly pathogenic AI when allowed to persist in a population [29].

There are challenges associated with the increase in popularity of free range poultry production; such as the greater production losses experienced compared to other farm types. A study in Australia found free range meat chicken performance had slower growth, higher mortalities and deteriorating feed conversion efficiency over time compared to non-free range chickens [30]. Similarly, a study that reviewed the levels of mortality in hens in different farm types in Great Britain found that the average on-farm mortalities during the laying period were 5.4%, 8.6% and 9.5% in cage, barn and free range layer farms respectively [31]. Further research on free range chicken farms in general needs to be performed in order to find ways to counteract these production issues, especially given the growing significance of this type of production in Australia.

Farmers in this study reported a lower proportion of birds using the range in meat chicken free range farms compared to layer free range farms. A higher variability was observed in responses from layer farms, with some indicating that 100% of birds use the range, when compared to meat chicken farms, with no one reporting a proportion of birds using the range higher than 50%. This lower proportion of birds that use the range in meat chicken farms may be in part due to the health related problems associated with a rapid growth rate of meat chickens, which cause birds to spend more time sitting on the floor and less time walking [32]. Such health conditions are caused by the large body weights and include increased heart abnormalities, tendon degeneration, varus/valgus deformations and femoral head necrosis [22, 27, 32].

Range use by birds has also been shown to be affected by outside temperature, flock size, precipitation and season. Few studies have reported the proportion of birds accessing the range in commercial-sized flocks, although it is generally agreed that larger flock sizes have a lower use of the range [33]. One study in Australia reported only 4% of layer hens in free range layer farms use the range in a flock of 16,000 hens [1], a much lower proportion than those reported by participant farms in this study. Certainly management practices limit range use by chickens on Australian meat chicken farms, as birds are not granted access until 21 days of age, and then access only permitted when climatic conditions are favorable, and depopulation begins roughly at 30 days of age. The limited time allowed outside with the rapid growth rates potentially poses a welfare problem. To counteract this welfare issue, slower-growing meat chicken breeds such as the Ross Rowan and Cobb-Sasso, have been developed and started to be used in the EU and USA. Such breeds take 50 to 100% longer to reach target weights compared to the fast-growing counterparts [22]. It is difficult to predict whether or not the development and use of slower-growing meat chicken breeds will become popular among producers in the Australian industry.

Another aspect investigated in this study was the percentage of the range being used by birds, with meat chicken farms reporting a higher percentage of the range being used compared to layer farms. One reason of this difference could be the smaller size of ranges in meat chicken farms when compared to those on layer farms, with meat chickens being able to easily disperse over the whole range area. Another reason may be a greater uniform spread of shade on the ranges of meat chicken farms compared to layer farms, reported in this study. The results in this study revealed trees and artificial shade structures being scattered and uniformly distributed, respectively, on a greater proportion of meat chicken farms compared to layer farms. This hypothesis aligns with a previous study, which found that the addition of tree cover increased the number of meat chickens that used the range area [34]. Similarly, other studies demonstrated that the addition of shelters on the range increased the length of time meat chickens were on the range [35, 36]. The preference of chickens to seek shaded areas is likely due to the fact that the ancestor of the domestic chicken is the red jungle fowl, which lived in an environment with extensive shade [1].

The median range stocking density for free range layer farms in this study was found to be approximately 6,500 birds per hectare and varied to over 20,000 birds per hectare on some farms. Australia has experienced political debate on the legal definition of free range eggs in recent years. Shortly after the completion of the farm surveys, Australia’s consumer affairs minister adopted a legally binding standard that defines free range eggs from farms with a stocking density on the range of up to 10,000 birds per hectare in 2016 [37]. Prior to this, there was only a voluntary Model Code of Practice published by the Commonwealth Scientific and Industrial Research Organisation (CSIRO) in 2002 which recommended a maximum of 1500 layer hens per hectare [38]. The range stocking densities of some of the free range layer farms visited may have therefore changed since completion of the on-farm interview. During these political debates, pop-hole requirements for free range farms appear to have remained unchanged. These are required through farm assurance schemes, but all schemes appear to use the same minimum standards. This minimum standard has been made for free range layer farms but is also commonly used as the minimum standard by meat chicken farms [39].

Another concern linked to the expansion of free range poultry production is the increasing environmental damage. Free range layer farms are thought to have greater environmental impact than free range meat chicken farms due to the long-lived nature and greater mobility of the layer bird compared to the meat chicken bird. In this study, the level of pasture on the range areas were, in general, lower in free range layer farms compared to meat chicken farms. In addition, pasture was completely bare next to the sheds in the majority (92%) of free range layer farms. The Australian Egg Corporation Limited (AECL) has created environmental guidelines to assist egg producers in reducing their environmental impact. Such recommendations include regular rotation of range areas to avoid excessive nutrient accumulation [40]. More than half of free range layer farms performed pasture rotation in this study. A report containing recommendations for meat chicken farms has also been created by the Rural Industries Research and Development Corporation (RIRDC). Such recommendations include maintaining adequate vegetation in free range areas to prevent soil erosion and nutrient loss from surface runoff [41].

In summary, this study addresses in part the lack of literature available detailing the descriptions of current farm designs and management practices in Australia for both layer and meat chicken farms of all types; cage, barn and free range. Obtaining a better understanding of these variations in an Australian context is useful in many aspects, including improving bird health and welfare management, environmental protection, disease prevention and preparedness, consumer education and communication with industry.

## Supporting information

S1 Text Avian influenza risk mitigation project in Australian commercial chicken farms—main questionnaire. (DOCX)

S2 Text Avian influenza risk mitigation project in Australian commercial chicken farms—biosecurity questionnaire. (DOCX)

## References

[1] Singh M, Cowieson A. Range use and pasture consumption in free-range poultry production. Animal Production Science. 2013;53:1202–8.

[2] AECL (Australian Egg Corporation Limited). Australian Egg Corporation Limited Annual Report 2015. North Sydney, NSW: Australian Egg Corporation; 2015.

[3] Animal Welfare: Commission report confirms the potential benefits of banning conventional battery cages for laying hens [Internet]. Brussels, Hungary: European Commission; 2008. Available from: http://europa.eu/rapid/press-release_IP-08-19_en.htm

[4] DEFRA (Department for Environment Food and Rural Affairs). United Kingdom Egg Statistics—Quarter 4, 2015. London, United Kingdom: National Statistics; 2016.

[5] Zhao Y, Shepherd T, Swanson J, Mench J, Karcher D, Xin H. Comparative evaluation of three egg production systems: Housing characteristics and management practices. Poultry Science. 2015;94:475–94. doi: 10.3382/ps/peu077 25737566 10.3382/ps/peu077 PMC4990892

[6] ACMF (Australian Chicken Meat Federation). The Australian Chicken Meat Industry: An Industry in Profile. North Sydney, NSW: Australian Chicken Meat Federation (ACMF) Inc; 2011.

[7] Sheppard A. The Structure and Economics of Broiler Production in England. England, UK: University of Exeter; 2004.

[8] Crane R, Laney S. Farm Business Survey 2013/2014 Poultry Production in England. Reading, UK: University of Reading; 2015.

[9] Hoyle P, Knox B, Crane R. Farm Business Survey 2014/15 Poultry Proudction in England. Reading, UK: University of Reading; 2016.

[10] National Chicken Council (NCC). Chickopedia: What Consumers Need to Know 2012 [12 July 2016]. Available from: http://www.nationalchickencouncil.org/about-the-industry/chickopedia/#four.

[11] Fanatico A, Owens C, Emmert J. Organic poultry production in the United States: Broilers. The Journal of Applied Poultry Research. 2009;18(2):355–66.

[12] Scott P, Turner A, Bibby S, Chamings A. Structure and Dynamics of Australia's Commercial Poultry and Ratite Industries. In: Department of Agriculture Fisheries and Forestry (DAFF), editor. Moonee Ponds, Victoria: Scolexia Animal and Avian Health Consultancy; 2009.

[13] Australian egg industry overview- June 2015 [Internet]. North Sydney, NSW: Australian Egg Corporation Limited; 2015

[14] NSW poultry egg industry overview 2015 [Internet]. Orange, NSW: New South Wales Department of Primary Industries; 2015; [23]

[15] NSW OEH (NSW Government Office of Environment and Heritage). Sydney Basin Bioregion 2011 [12 January 2016]. Available from: http://www.environment.nsw.gov.au/bioregions/SydneyBasinBioregion.htm.

[16] New South Wales Government. Swift action eradicates Avian Influenza at Maitland egg farm. In: Department of Primary Industries, editor. 2012.

[17] BOM (Bureau of Meterology). Weather Station Directory 2016 [4 February 2016]. Available from: http://www.bom.gov.au/climate/data/stations/.

[18] AECL (Australian Egg Corporation Limited). Australian Egg Industry Overview December 2014 2015 [12 January 2015]. Available from: https://www.aecl.org/resources/industry-statistics/.

[19] The Australian Chicken Meat Industry: An Industry in Profile [Internet]. North Sydney, NSW: Australian Chicken Meat Federation (ACMF) Inc; 2013; [23 October 2015]. Available from: http://www.chicken.org.au/page.php?id=2

[20] NSW DPI (New South Wales Department of Primary Industries). Avian Influenza 2014 [cited 7 March 2015]. Available from: http://www.dpi.nsw.gov.au/agriculture/livestock/poultry/health-disease/avian-influenza/more/questions-answers#Are-there-any-avian-influenza-viruses-present-in-Australia?

[21] Department of Agriculture Fisheries and Forestry (DAFF). National Farm Biosecurity Manual Poultry Production. 1st ed. ACT: Commonwealth of Australia; 2009.

[22] Robins A, Phillips C. International approaches to the welfare of meat chickens. World's Poultry Science Journal. 2011;67:351–69.

[23] Thomas D, Ravindran V, Thomas D, Camden B, Cottam Y, Morel P, et al Influence of stocking density on the performance, carcass characteristics and selected welfare indicators of broiler chickens. New Zealand Veterinary Journal. 2004;52(2):76–81. doi: 10.1080/00480169.2004.36408 15768100 10.1080/00480169.2004.36408

[24] Feddes J, Emmanuel E, Zuidof M. Broiler Performance, Bodyweight Variance, Feed and Water Intake, and Carcass Quality at Different Stocking Densities. Poultry Science. 2002;81(6):774–9. 12079042 10.1093/ps/81.6.774

[25] Martrenchar A, Morisse J, Huonnic D, Cotte J. Influence of stocking density on some behavioural, physiological and productivity traits of broilers. Veterinary Research. 1997;28:473–80. 9342823

[26] Bilgili S. The Risks and Benefits of Flock Thinning. Auburn University,. Department of Poultry Science: Auburn, Alabama, USA 2017.

[27] Barnes H. Other Bacterial Diseases In: Saif Y, Fadly A, Glisson J, McDougald L, Nolan L, Swayne D, editors. Diseases of Poultry. 12 ed. Iowa USA: Blackwell Publishing; 2009 p. 941–51.

[28] East IJ. Adoption of biosecurity practices in the Australian poultry industries. Australian Veterinary Journal. 2007;85(3):107–12. doi: 10.1111/j.1751-0813.2007.00113.x 17359311 10.1111/j.1751-0813.2007.00113.x

[29] Swayne DE. Avian Influenza. First ed. Iowa USA: Blackwell Publishing; 2008.

[30] Durali T, Groves P, Cowieson A, editors. Comparison of Performance of Commercial Conventional and Free Range Broilers. Australian Poultry Science Symposium; 2012; Sydney.

[31] Weeks C, Brown S, Richards G, Wilkins L, Knowles T. Levels of mortality in hens by end of lay on farm and in transit to slaughter in Great Britain. The Veterinary Record. 2012;647(170 25). doi: 10.1136/vr.100728 22678619 10.1136/vr.100728

[32] Hocking P. Unexpected consequences of genetic selection in broilers and turkeys: problems and solutions. British Poultry Science. 2014;55(1):1–12. doi: 10.1080/00071668.2014.877692 24397366 10.1080/00071668.2014.877692

[33] Hartcher K, Hickey K, Hemsworth P, Cronin G, Wilkinson S, Singh M. Relationships between range access as monitored by radio frequency identification technology, fearfulness, and plumage damage in free-range laying hens. 2016. 2016;10(5):847–53. doi: 10.1017/S1751731115002463 26593871 10.1017/S1751731115002463

[34] Lubac S, Mirabito L. Relationship between activies of 'Red label' type chickens in an outdoor run and external factors. British Poultry Science. 2001;42(S14–S15).

[35] Zeltner E, Hirt H. Factors involved in the improvement of the use of hen runs. Applied Animal Behaviour Science. 2008;114:395–408.

[36] Dawkins M, Cook P, Whittingham M, Mansell K, Harper A. What makes free-range broiler chickens range? In situ measurement of habitat preference. Animal Behaviour. 2003;66:151–60.

[37] Consumer Affairs Australian New Zealand. Decision Regulation Impact Statement on Free Range Egg Labelling. In: Cabinet DotPMa, editor. Parkes, ACT: Australian Government The Treasury; 2016.

[38] CSIRO (The Commonwealth Scientific and Industrial Research Organisation). Primary Industries Standing Committee Model Code of Practice for the Welfare of Animals. 4th ed. Collingwood, Victoria: CSIRO Publishing; 2002.

[39] Egg Standards Australia. Farm Standard for Egg Producers: Australian Egg Corporation Limited; 2012.

[40] McGahan E, Barker S, Tucker R. Environmental Guidelines for the Australian Egg Industry. North Sydney, NSW: Australian Egg Corporation Limited; 2008.

[41] McGahan E, Bielefeld N, Widemann S, Keane O. National Environmental Management System for the Meat Chicken Industry—Version 2. Barton, ACT: Rural Industries Research and Development Corporation; 2014.

