# Correction: Comparisons of management practices and farm design on Australian commercial layer and meat chicken farms: Cage, barn and free range

**DOI:** 10.1371/journal.pone.0194086

**Published:** 2018-03-05

**Authors:** Angela Bullanday Scott, Mini Singh, Jenny-Ann Toribio, Marta Hernandez-Jover, Belinda Barnes, Kathryn Glass, Barbara Moloney, Amanda Lee, Peter Groves

There is an error in the third sentence of the Abstract. The correct sentence is: This study aimed to describe features of Australian commercial chicken farms, with particular interest in free range farms, by conducting on-farm interviews of 25 free range layer farms, nine cage layer farms, nine barn layer farms, 15 free range meat chicken farms and 15 barn meat chicken farms in the Sydney basin bioregion and South East Queensland.
