# Supplementary material for: Comparisons of management practices and farm design on Australian commercial layer and meat chicken farms: Cage, barn and free range
Source: PLoS One. 2017 Nov 22;12(11):e0188505. doi: 10.1371/journal.pone.0188505 (PMC5699831; doi:10.1371/journal.pone.0188505)
Supplement: S1 Text — (DOCX) [file pone.0188505.s001.docx]

Poultry Research Foundation

Faculty of Veterinary Science

JL Shute Building C01, 425 Werombi Rd

Camden NSW 2570

**Personal Information**

**_____________________________________________________________________________________________**

| Farm owner |  | | | Street Address |  | | |
| --- | --- | --- | --- | --- | --- | --- | --- |
|  | | |  |  | | |  |
| Farm manager |  | | | Suburb & postcode | |  | |
|  | | |  |  | | |  |
| Company |  | | | Property Identification code (PIC) | | |  |
|  | | |  |  | | |  |
| Phone/mobile no | |  | | | | |  |
|  | | |  |  | | |  |
| Email address | |  | | | | |  |
|  | | |  |  | | |  |
| Farm website | |  | | | | |  |

**Farm Information**

**_____________________________________________________________________________________________**

| 1. Is this farm a farm of several under the same ownership? | | | | | | | | | | | | | | | | | | | | | Yes | | |  | | No | |  | |  |  |  |
| --- | --- | --- | --- | --- | --- | --- | --- | --- | --- | --- | --- | --- | --- | --- | --- | --- | --- | --- | --- | --- | --- | --- | --- | --- | --- | --- | --- | --- | --- | --- | --- | --- |
|  | | | | | | | | | | | | | | | | | | | | |  | | |  | |  | |  | |  |  |  |
| 1. How many farms do you operate? | | | | | | | | | | |  | | | | | |  |  |  |  |  |  |  |  |  |  |  |  |  |  |  |  |
|  | | | | | | | | | | | | | | | | | | | |  | | | | | | | | | |  |  |  |
| 1. Where are the locations of the other farms? | | | | | | | | | | | | | | | | | | | |  | | | | | | | | | |  |  |  |
|  | | | | | | | | | | | | | | | | | | | | | | | | | | | | | | | | |
|  | | | | | | | | | | | | | | | | | | | |  | | | | | | | | | | | | |
| 1. How many chickens are there on this farm? | | | | | | | | | | | | | |  | | | | | | | | | | | | | | | | | | |
|  | | | | | | | | | | | | | |  | | | | | | | | | | | | | | | | | | |
| 1. How many sheds are on the farm? | | | | | | | | | | | | | |  | | | | | | | | | | | | | | | | | | |
|  | | | | | | | | | | | | | |  | | | | | | | | | | | | | | | | | | |
| 1. How many chickens are there per shed on this farm? | | | | | | | | | | | | | |  | | | | | | | | | | | | | | | | | | |
|  | | | | | | | | | | | | | | | | | | | | | | | | | | | | | | | | |
| 1. What are the shed dimensions (height x width x length in meters)? | | | | | | | | | | | | | | | | | | | | | | | | | | | | | | | | |
|  | | | | | | | | | | | | | | | | | | | | | | | | | | | | | | | | |
|  | | | | | | | | | | | | | | | | | | | | | | | | | | | | | | | | |
|  | | | | | | | | | | | | | | | | | | | |  | | | | | | | | | | | | |
| 1. What breed of chickens is kept on this farm? | | | | | | | | | | | | | | | | | | | | | | | | | | | | | | | | |
|  | | | | | | | | | | | | | | | | | | | | | | | | | | | | | | | | |
| Isabrown | | | | | | |  | | | Hyline | | | | | | | | | | | |  | Rhode Island Red | | | | | | | |  | |
| Bonds | | | | | | |  | | | Lohmann | | | | | | | | | | | |  | Ross | | | | | | | |  | |
| Cobb | | | | | | |  | | | Other | | | | | | | | | | | |  | | | | | | | | | | |
|  | | | | | | | | | | | | | | | | | | | |  | | | | | | | | | | | | |
|  | | | | | | | | | | | | | | | | | | | | | | | | | | | | | | | | |
| 1. How many flocks (i.e. birds of different age groups) do you have on this farm? | | | | | | | | | | | | | | | | | | | | | | | | | | | | | | | | |
|  | | | | | | | | | | | | | | |  | | | | | | | | | | | | | | | | | |
|  | | | | | | | | | | | | | | | | | | | | | | | | | | | | | | | | |
| 1. Describe the proportion of different age groups of chickens on the farm and if there are any changes with these proportions over time (e.g. 20% of birds on the farm are over 40wks old now but in summer this changes to 10%). | | | | | | | | | | | | | | | | | | | | | | | | | | | | | | | | |
|  | | | | | | | | | | | | | | | | | | | | | | | | | | | | | | | | |
|  | | | | | | | | | | | | | | | | | | | | | | | | | | | | | | | | |
| 1. Are birds of different age groups completely separated or are there interactions between different bird groups? | | | | | | | | | | | | | | | | | | | | | | | | | | | | | | | | |
| Separated | | | |  | | | | Interactions | | | | | | | | |  | | |  |  |  |  |  |  |  |  |  |  |  |  |  |
|  | | | | | | | | | | | | | | | | |  | | | | | | | | | | | | | | | |
| 1. At what age are the chickens when flock depopulation/ days to processing occurs? | | | | | | | | | | | | | | | | | | | | | | | | | | | | |  | | | |
|  | | | | | | | | | | | | | | | | | | | |  | | | | | | | | | | | | |
| 1. Are all birds removed at once? | | | | | | | | | | | | | Yes | | | | |  | | | No | | | |  | |  |  |  |  |  |  |
|  | | | | | | | | | | | | | | | | | | | |  | | | | | | | | | | | | |
| 1. If not, describe the ‘thinning out’ procedure. | | | | | | | | | | | | | | | | | | | | | | | | | | | | | | | | |
|  | | | | | | | | | | | | | | | | | | | | | | | | | | | | | | | | |
|  | | | | | | | | | | | | | | | | | | | |  | | | | | | | | | | | | |
| 1. What is the average turnaround time (i.e. length of time a shed is empty between when birds are removed and the replacement of new stock)? | | | | | | | | | | | | | | | | | | | | | | | | | | | | | | | | |
|  | | | | | | | | | | | | | | | | | | | |  | | | | | | | | | | | | |
|  | | | | | | | | | | | | | | | | | | | | | | | | | | | | | | | | |
| 1. Where is feed stored? | | | | | | | | | | | | | | | | | | | |  | | | | | | | | | | | | |
| Bagged inside | | | | |  | | | Bagged outside | | | | | | | |  | | | Uncovered silos | | | | | | | |  | Covered silos | | | |  |
| Other | | |  | | | | | | | | | | | | | | | | | | | | | | | | | | | | | |
|  | | | | | | | | | | | | | | | | | | | |  | | | | | | | | | | | | |
| 1. What environmental control methods are there in the sheds? | | | | | | | | | | | | | | | | | | | | | | | | | | | | | | | | |
| Foggers/ sprinklers | | | | | | |  | | | Fans | | | | | | | | | | | |  | Side curtains | | | | | | | |  | |
| Cooling pads | | | | | | |  | | | Tunnel Ventilation | | | | | | | | | | | |  | Heater | | | | | | | |  | |
| Other | | |  | | | | | | | | | | | | | | | | | | | | | | | | | | | | | |
|  | | | | | | | | | | | | | | | | | | | |  | | | | | | | | | | | | |
| 1. What type and how many drinkers are there per shed? | | | | | | | | | | | | | | | | | | | |  | | | | | | | | | | | | |
| Bells | | | | | | |  | | | Nipples | | | | | | | | | | | |  | Troughs | | | | | | | |  | |
| Other | | |  | | | | | | | | | | | | | | | | | | | | | | | | | | | | | |
| Comments | | |  | | | | | | | | | | | | | | | | | | | | | | | | | | | | | |
|  | | | | | | | | | | | | | | | | | | | |  | | | | | | | | | | | | |
| 1. What type and how many feeders are there per shed? | | | | | | | | | | | | | | | | | | | |  | | | | | | | | | | | | |
| Gravity pan | | | | | | |  | | | Automatic pan | | | | | | | | | | | |  | Troughs | | | | | | | |  | |
| Chain feeder | | | | | | |  | | |  |  |  |  |  |  |  |  |  |  |  |  |  |  |  |  |  |  |  |  |  |  |  |
| Other | | |  | | | | | | | | | | | | | | | | | | | | | | | | | | | | | |
| Comments | | |  | | | | | | | | | | | | | | | | | | | | | | | | | | | | | |
|  | | | | | | | | | | | | | | | | | | | |  | | | | | | | | | | | | |
| 1. What type and how many nests are there per shed? | | | | | | | | | | | | | | | | | | | |  | | | | | | | | | | | | |
| Wooden boxes | | | | | | |  | | | Cut out drum | | | | | | | | | | | |  | Rollaway nest boxes | | | | | | | |  | |
| Other | | |  | | | | | | | | | | | | | | | | | | | | | | | | | | | | | |
| Comments | | |  | | | | | | | | | | | | | | | | | | | | | | | | | | | | | |
|  | | |  | | | | | | | | | | | | | | | | | | | | | | | | | | | | | |
| 1. For layers- what is the average egg production (number of eggs laid per hen per week)? | | | | | | | | | | | | | | | | | | | | | | | | | | | |  | | | | |
|  | | | | | | | | | | | | | | | | | | | | | | | | | | | | | | | | |
| 1. For broilers- what is the average growth rate? E.g. number of weeks to meet a certain weight (what weight do you aim for)? Or feed conversion ratio (FCR) if known? | | | | | | | | | | | | | | | | | | | | | | | | | | | | | | | | |
|  | | | | | | | | | | | | | | | | | | | | | | | | | | | | | | | | |
|  | | | | |  |  |  |  |  |  |  |  |  |  |  |  |  |  |  |  |  |  |  |  |  |  |  |  |  |  |  |  |
|  | | | | | | | | | | | | | | | | | | | | | | | | | | | | | | | | |
| 1. Is surface water (e.g. dams, creeks) present on the property? If yes, what type and how many? | | | | | | | | | | | | | | | | | | | | | | | | | | | | | | | | |
|  | | | | | | | | | | | | | | | | | | | | | | | | | | | | | | | | |
| Yes |  | No | |  | |  | | | Comments | | |  | | | | | | | | | | | | | | | | | | | | |
|  |  |  |  |  | |  |  |  |  |  |  |  |  |  |  |  |  |  |  |  |  |  |  |  |  |  |  |  |  |  |  |  |
|  | | | | | | | | | | | | | | | | | | | | | | | | | | | | | | | | |
| 1. Is surface water present outside but nearby the property? If yes, what type (e.g. river, lake, wetlands)? How far away from the property? | | | | | | | | | | | | | | | | | | | | | | | | | | | | | | | | |
|  | | | | | | | | | | | | | | | | | | | | | | | | | | | | | | | | |
| Yes |  | No | |  | |  | | | Comments | | |  | | | | | | | | | | | | | | | | | | | | |
|  |  |  |  |  | |  |  |  |  |  |  |  |  |  |  |  |  |  |  |  |  |  |  |  |  |  |  |  |  |  |  |  |
|  |  |  |  |  | |  |  |  |  |  |  |  |  |  |  |  |  |  |  |  |  |  |  |  |  |  |  |  |  |  |  |  |

**Water**

**_____________________________________________________________________________________________**

| 1. For free range producers- do you irrigate the range? | | | | | | | | | | | | | | | | | | | | | | | | Yes | | | |  | | No | |  |
| --- | --- | --- | --- | --- | --- | --- | --- | --- | --- | --- | --- | --- | --- | --- | --- | --- | --- | --- | --- | --- | --- | --- | --- | --- | --- | --- | --- | --- | --- | --- | --- | --- |
|  | | | | | | | | | | | | | | | | | | | | | | | | | | | | | | | | |
| 1. What is the source of the drinking water for the chickens? | | | | | | | | | | | | | | | | | | | | | | | | | | | | | | | | |
|  | | | | | | | | | | | | | | |  | | | | | | | | | | | | | | | | | |
| Farm dam | | | | | | | | |  | | Nearby natural water body | | | | | | | |  | | | Town water | | | | | | | | | |  |
| Rain water | | | | | | | | |  | | Bore water | | | | | | | |  | | |  |  |  |  |  |  |  |  |  |  |  |
| Other | | |  | | | | | | | | | | | | | | | | | | | | | | | | | |  |  |  |  |
|  | | | | | | | | | | | | | | |  | | | | | | | | | | | | | | | | | |
| 1. Is this source the same for the foggers, sprinklers, cooling pads and irrigation of the range? | | | | | | | | | | | | | | | | | | | | | | | | | | | Yes | |  | | No |  |
|  |  |  |  |  |  |  |  |  |  |  |  |  |  |  |  |  |  |  |  |  |  |  |  |  |  |  |  |  |  | |  |  |
|  |  |  |  |  |  |  |  |  |  |  |  |  |  |  |  |  |  |  |  |  |  |  |  |  |  |  |  |  |  | |  |  |
|  | | | | | | | | | | | | | | | | | | | | | | | | | | | | | | | | |
| 1. If no, what is the source of water for the foggers, sprinklers, cooling pads and irrigation of the range? | | | | | | | | | | | | | | | | | | | | | | | | | | | | | | | | |
|  | | | | | | | | | | | | | | |  | | | | | | | | | | | | | | | | | |
| Farm dam | | | | | | | | |  | | Nearby natural water body | | | | | | | |  | | | Town water | | | | | | | | | |  |
| Rain water | | | | | | | | |  | | Bore water | | | | | | | |  | | |  |  |  |  |  |  |  |  |  |  |  |
| Other | |  | | | | | | | | | | | | | | | | | | | | | | | | | | | | | | |
| Comments | |  | | | | | | | | | | | | | | | | | | | | | | | | | | | | | | |
|  | | | | | | | | | | | | | | | | | | | | | | | | | | | | | | | | |
| 1. Is the water treated? | | | | | | | | | | | | | | | | | | | | | | | | | | | | | | | | |
|  | | | | | | | | | | | | | | |  | | | | | | | | | | | | | | | | | |
| Yes |  | | | Yes- drinking water only | | | | | | | | |  | Yes- drinking water and foggers/ sprinklers, cooling pads | | | | | | | | | | | | | | | | | |  |
| No |  | | |  | | | | | | | | | | | | | | | | | | | | | | | | | | | | |
|  |  | |  | | | | | | | | | | | | | | | | | | | | | | | | | | | | | |
| 1. If yes, what method do you use to treat the water? | | | | | | | | | | | | | | | | | | | | | | | | | | | | | | | | |
|  | | | | | | | | | | | | | | | | |  | | | | | | | | | | | | | | | |
| Chlorination | | | | |  | |  | | | | | Phosphoric acid | | | | |  | | |  | | | | | | UV light | | | | | |  |
| Other | | | | |  | | | | | | | | | | | | | | | | | | | | | | | | | | | |
|  | | | | | | | | | | | | | | | | | | | | | | | |  | | | |  | |  | |  |
| 1. Is the treatment process automatic or manual? | | | | | | | | | | | | | | | | | | | | | | | Automatic | | | | |  | | Manual | |  |
|  | | | | | | | | | | | | | | | | | | | | | | | | | | | | | | | | |
| 1. How often is the treatment process performed? | | | | | | | | | | | | | | |  | | | | | | | | | | | | | | | | | |
|  | | | | | | | | | | | | | | | | | | | | | | | |  | | | |  | |  | |  |
| 1. Do you test to make sure treatment is successful? | | | | | | | | | | | | | | | | | | | | | | | | Yes | | | |  | | No | |  |
|  | | | | | | | | | | | | | | | | | | | | | | | | | | | | | | | | |
| 1. If yes, what method do you use to test for successful treatment and how often is it performed? | | | | | | | | | | | | | | | | | | | | | | | | | | | | | | | | |
|  | | | | | | | | | | | | | | |  | | | | | | | | | | | | | | | | | |
| Chlorination strips | | | | | |  | |  | | Commercial testing kit | | | | | |  | |  | | | Other | | | |  | | | | | | | |
| Comments | | |  | | | | | | | | | | | | | | | | | | | | | | | | | | | | | |

**Health**

**_____________________________________________________________________________________________**

| 1. How often are the flocks inspected? | | | | | | | | | | | | | | | | | | | | | | | |  |
| --- | --- | --- | --- | --- | --- | --- | --- | --- | --- | --- | --- | --- | --- | --- | --- | --- | --- | --- | --- | --- | --- | --- | --- | --- |
|  | | | | | | | | | | | | | | | | | | | | | | | |  |
| Daily |  | | Twice a day | | | | | | | | |  | | Three times a day | | | | | | | |  | |  |
| Other |  | | | | | | | | | | | | | | | | | | | | | | |  |
|  | | | | | | | | | | | | | | | | | | | | | | | |  |
| 1. Do you keep flock health records? | | | | | | | | | | | | | | | | | | Yes |  | No | | |  |  |
|  | | | | | | | | | | | | | | | | | | | | | | | |  |
| 1. What type of information do you record on these? | | | | | | | | | | | | | | | | | | | | | | | | |
| Vaccination | | | | |  | Egg production | | | | | | | | |  | Feed conversion ratio (FCR) | | | | | |  | | |
| Weight | | | | |  | Sick birds | | | | | | | | |  | Mortalities | | | | | |  | | |
| Medications given | | | | |  | Other | |  | | | | | | | | | | | | | | | | |
|  | | | | | | | | | | | | | | | | | | | | | | | | |
| 1. What would you consider as unusual signs in your birds? | | | | | | | | | | | | | | | | | | | | | | | | |
| Drop in egg production | | | | |  | Slow growth rate | | | | | | | | |  | Lethargy, quietness | | | | | |  | | |
| Respiratory signs (sneezing) | | | | |  | Reduced feed intake | | | | | | | | |  | Eye signs (tearing, swelling) | | | | | |  | | |
| Increased deaths | | | | |  | Gastrointestinal signs (regurgitation, abnormal faeces) | | | | | | | | | | | | | | | |  | | |
| Other | | | | |  | | | | | | | | | | | | | | | | | | | |
|  | | | | | | | | | | | | | | | | | | | | | | | | |
| 1. What do you usually do when you notice unusual signs in your birds? | | | | | | | | | | | | | | | | | | | | | | | | |
| Give medication | | | | |  | Notify someone (not vet) | | | | | | | | |  | Notify vet | | | | | |  | | |
| Do nothing | | | | |  | Other | |  | | | | | | | | | | | | | | | | |
|  | | | | | | | | | | | | | | | | | | | | |  | | | |
| 1. What proportion of the flock affected by unusual signs do you consider important to notify someone? | | | | | | | | | | | | | | | | | | | | |  | | | |
|  | | | | | | | | | | | | | | | | | | | | | | | | |
| 1. Who do you notify? | | | | | | | | | | | | | | | | | | | | | | | | |
| Farm owner/ manager | | | | |  | Vet | | | | | | | | |  | Friend | | | | | |  | | |
| No one | | | | |  | Other | |  | | | | | | | | | | | | | | | | |
|  | | | | | | | | | | | | | | | | | | | | | | | | |
| 1. In the past year, what were/are the 3 main causes of mortality (e.g. cannibalism, predation, infectious disease)? On average, what percentage of the flock is/was affected? When did these happen? | | | | | | | | | | | | | | | | | | | | | | | | |
|  | | | | | | | | | | | | | | | | | | | | | | | | |
|  | | | | | | | | | | | | | | | | | | | | | | | | |
| 1. What other diseases (which do not necessarily cause mortality) are present on the property or have been? What percentage of the flock was affected? How long have and when were these diseases present? | | | | | | | | | | | | | | | | | | | | | | | | |
|  | | | | | | | | | | | | | | | | | | | | | | | | |
|  | | | | | | | | | | | | | | | | | | | | | | | | |
| 1. What 3 diseases of chickens (that may or may not have occurred on your farm) do you worry about the most? | | | | | | | | | | | | | | | | | | | | | | | | |
|  | | | | | | | | | | | | | | | | | | | | | | | | |
|  | | | | | | | | | | |  | | | | | | | | | | | | | |
| 1. How often does a veterinarian visit your farm? | | | | | | | | | | |  | | | | | | | | | | | | | |
|  | | | | | | | | | | | | | | | | | | | | | | | | |
| 1. What are the main reasons for the veterinarian’s visit? | | | | | | | | | | | | | | | | | | | | | | | | |
| Quality assurance | |  | | Disease investigation | | |  | | | Other | | |  | | | | | | | | | | | |
|  | | | | | | | | | | | | | | | | | | | | | | | | |
| 1. What is the name of the veterinarian? | | | | | | | | |  | | | | | | | | | | | | | | | |
|  | | | | | | | | | | | | | | | | | | | | | | | | |
| 1. What are the chickens vaccinated against? | | | | | | | | | | | | | | | | | | | | | | | | |
| Infectious bursal disease | | | | |  | Infectious bronchitis | | | | | | | | |  | Avian encephalomyelitis | | | | | |  | | |
| Newcastle disease | | | | |  | Marek’s disease | | | | | | | | |  | Infectious laryngotracheitis | | | | | |  | | |
| Fowl pox | | | | |  | Chicken anaemia virus | | | | | | | | |  | Egg drop syndrome ‘76 | | | | | |  | | |
| Mycoplasma Gallisepticum | | | | |  | Coccidia (Paracox) | | | | | | | | |  | Salmonella Typhimurium | | | | | |  | | |
| Mycoplasma Synoviae | | | | |  | Infectious Coryza | | | | | | | | |  | No vaccinations | | | | | |  | | |
| Unknown | | | | |  | Other | | | | | | | | |  | | | | | | | | | |
|  | | | | | | | | | | | | | | | | | | | | | | | | |
| 1. Do you have a vaccination plan? At what age are the chickens vaccinated? | | | | | | | | | | | | | | | | | | | | | | | | |
|  | | | | | | | | | | | | | | | | | | | | | | | | |
|  | | | | | | | | | | | | | | | | | | | | | | | | |
| 1. Who does the vaccination? | | | | | | | | | | | | | | | | | | | | | | | | |
| Self-administered | |  | | Vaccination crew | | |  | | | Other | | |  | | | | | | | | | | | |
|  | | | | | | | | | | | | | | | | | | | | | | | | |
| 1. Why do you vaccinate against Egg Drop Syndrome ’76? [Only ask if they are vaccinating for this] | | | | | | | | | | | | | | | | | | | | | | | | |
|  | | | | | | | | | | | | | | | | | | | | | | | | |
|  | | | | | | | | | | | | | | | | | | | | | | | | |
| 1. What signs in chickens do you consider as them being infected with avian influenza virus? | | | | | | | | | | | | | | | | | | | | | | | | |
| Drop in egg production | | | | |  | Slow growth rate | | | | | | | | |  | | Lethargy, quietness | | | | |  | | |
| Respiratory signs (sneezing) | | | | |  | Reduced feed intake | | | | | | | | |  | | Eye signs (tearing, swelling) | | | | |  | | |
| Increased deaths | | | | |  | Gastrointestinal signs (regurgitation, abnormal faeces) | | | | | | | | | | | | | | | |  | | |
| Other | | | | |  | | | | | | | | | | | | | | | | | | | |

**Range information (for free range producers)**

**_____________________________________________________________________________________________**

|  | | | | | | | | | | | | | | | | | |
| --- | --- | --- | --- | --- | --- | --- | --- | --- | --- | --- | --- | --- | --- | --- | --- | --- | --- |
| 1. What is the average range area (in hectares)? | | | | | | | |  | | | | | | | | | |
|  | | | | | | | | | | | | |  | | | | |
| 1. How do you manage run off from the sheds i.e. how is it collected (e.g. terminal pond) and how is it treated (e.g. vegetative strip)? | | | | | | | | | | | | | | | | | |
| Not collected or treated | | |  | Collected in pond/dam | | | | |  | | Collected in pond/dam and irrigated | | | | |  | |
| Treated in vegetative strip | | | | | |  | Both collected in pond and treated in vegetative strip | | | | | | | | |  | |
| Other |  | | | | | | | | | | | | | | | | |
|  | | | | | | | | | | | | |  | | | | |
| 1. What percentage of the flock uses the range? | | | | | | | | | | | | |  | | | | |
|  | | | | | | | | | | | | |  | | | | |
| 1. What percentage of the range is used by the birds? | | | | | | | | | | | | |  | | | | |
|  | | | | | | | | | | | | | | | | | |
| 1. At what times are birds allowed access to the range? | | | | | | | | | | | | | | | | | |
| 24 hour access | | |  | Sunrise to sunset | | | | |  | | Specific time of the day | | | | |  | |
| Other |  | | | | | | | | | | | | | | | | |
|  | | | | | | | | | | | |  | |  |  | |  |
| 1. Does this vary with season and weather? | | | | | | | | | | | | Yes | |  | No | |  |
| Comments |  | | | | | | | | | | | | | | | | |
|  | | | | | | | | | | | | |  | | | | |
| 1. At what age are birds allowed access to the range? | | | | | | | | | | | | |  | | | | |
|  | | | | | | | | | |  | | | | | | | |
| 1. How often do chickens escape the perimeter fencing? | | | | | | | | | |  | | | | | | | |
|  | | | | | | | | | | | |  | |  |  | |  |
| 1. Do the chickens have access to outside surface water on the range? | | | | | | | | | | | | Yes | |  | No | |  |
| Comments |  | | | | | | | | | | | | | | | | |
|  | | | | | | | | | | | | | | | | | |
| 1. Are there any ditches/holes/drains on the range that fill up with water when it rains/ during irrigation? | | | | | | | | | | | | | | | | | |
| Yes |  | No | |  |  |  |  |  |  |  |  |  |  |  |  |  |  |
| Comments |  | | | | | | | | | | | | | | | | |
|  | | | | | | | | | | | | | | | | | |
| 1. Do you offer any food or water out on the range? If yes, how often do you feed out on the range? | | | | | | | | | | | | | | | | | |
| Yes |  | No | |  |  |  |  |  |  |  |  |  |  |  |  |  |  |
| Comments |  | | | | | | | | | | | | | | | | |
|  | | | | | | | | | | | | | | | | | |
| 1. Do you clean the range area? What methods do you perform (e.g. mowing, manure removal)? How often do you perform this? | | | | | | | | | | | | | | | | | |
| Yes |  | No | |  |  |  |  |  |  |  |  |  |  |  |  |  |  |
| Comments |  | | | | | | | | | | | | | | | | |

**Contact with wild birds and other animals**

**_________________________________________________________________________________________________________________________________**

|  | **Nearby surface water** | | | | | | | | **Inside sheds** | | | | | | | | **In feed storage areas** | | | | | | | | **On range** | | | | | | | |
| --- | --- | --- | --- | --- | --- | --- | --- | --- | --- | --- | --- | --- | --- | --- | --- | --- | --- | --- | --- | --- | --- | --- | --- | --- | --- | --- | --- | --- | --- | --- | --- | --- |
|  |  | | | | | | | |  | | | | | | | |  | | | | | | | |  | | | | | | | |
| **Wild birds** |  | | | | | | | |  | | | | | | | |  | | | | | | | |  | | | | | | | |
| 1. Have you observed any wild birds in these areas? |  | | Yes | |  | |  | |  | | Yes | |  | |  | |  | | Yes | |  | |  | |  | Yes | | |  | |  | |
|  |  |  | No | |  | |  |  |  |  | No | |  | |  |  |  |  | No | |  | |  |  |  | No | | |  | |  |  |
|  |  |  |  |  |  | |  |  |  |  |  |  |  | |  |  |  |  |  |  |  | |  |  |  |  |  |  |  | |  |  |
| 1. If yes, what species? | Waterfowl (ducks, geese, swans) | | | | | | |  | Waterfowl (ducks, geese, swans) | | | | | | |  | Waterfowl (ducks, geese, swans) | | | | | | |  | Waterfowl (ducks, geese, swans) | | | | | | |  |
|  |  |  |  |  |  |  |  |  |  |  |  |  |  |  |  |  |  |  |  |  |  |  |  |  |  |  |  |  |  |  |  |  |
|  | Shorebirds (gulls, waders, plovers) | | | | | | |  | Shorebirds (gulls, waders, plovers) | | | | | | |  | Shorebirds (gulls, waders, plovers) | | | | | | |  | Shorebirds (gulls, waders, plovers) | | | | | | |  |
|  |  |  |  |  |  |  |  |  |  |  |  |  |  |  |  |  |  |  |  |  |  |  |  |  |  |  |  |  |  |  |  |  |
|  | Other | | | | | | |  | Other | | | | | | |  | Other | | | | | | |  | Other | | | | | | |  |
|  | None | | | | | | |  | None | | | | | | |  | None | | | | | | |  | None | | | | | | |  |
|  |  | | | | | | |  |  | | | | | | | |  | | | | | | | |  | | | | | | | |
|  |  | | | | | | |  |  | | | | | | | |  | | | | | | | |  | | | | | | | |
| 1. How often do they visit? Are they more frequent during a certain time of the day or year? |  | | | | | | | |  | | | | | | | |  | | | | | | | |  | | | | | | | |
|  |  | | | | | | | |  | | | | | | | |  | | | | | | | |  | | | | | | | |
| 1. What interactions do they have with poultry? | Direct (physical) contact | | | | | | |  | Direct (physical) contact | | | | | | |  | Direct (physical) contact | | | | | | |  | Direct (physical) contact | | | | | | |  |
|  |  |  |  |  |  |  |  |  |  |  |  |  |  |  |  |  |  |  |  |  |  |  |  |  |  |  |  |  |  |  |  |  |
|  | Indirect contacting (perching in shed, eating feed, drinking water) | | | | | | |  | Indirect contacting (perching in shed, eating feed, drinking water) | | | | | | |  | Indirect contacting (perching in shed, eating feed, drinking water) | | | | | | |  | Indirect contacting (perching in shed, eating feed, drinking water) | | | | | | |  |
|  |  |  |  |  |  |  |  |  |  |  |  |  |  |  |  |  |  |  |  |  |  |  |  |  |  |  |  |  |  |  |  |  |
|  |  |  |  |  |  |  |  |  |  |  |  |  |  |  |  |  |  |  |  |  |  |  |  |  |  |  |  |  |  |  |  |  |
|  | None | | | | | | |  |  | | | | | | | | None | | | | | | |  |  | |  |  | |  | | |
|  |  |  | |  | |  | | |  |  | |  | |  | | |  |  | |  | |  | | |  | |  |  | |  | | |
| 1. Do they frequently visit during feed spills? |  |  | |  | |  | | |  |  | | | | | | |  | Yes | |  | |  | | |  | |  |  | |  | | |
|  |  |  | |  | |  |  |  |  |  |  |  |  |  |  |  |  | No | |  | |  |  |  |  |  |  |  | |  |  |  |
|  |  | | | | | | | |  | | | | | | | |  | | | | | | | |  | | | | | | | |

|  | **Large mammals (e.g. foxes, cats)** | | **Small mammals (e.g. rodents)** | | | | **Reptiles** | | | **Insects, spiders** | | | **Other** | | |
| --- | --- | --- | --- | --- | --- | --- | --- | --- | --- | --- | --- | --- | --- | --- | --- |
| **Other wild animals** |  | |  | | | |  | | |  | | |  | | |
|  |  |  |  |  |  |  |  |  |  |  |  |  |  | | |
| 1. Have you observed any other wild creatures (besides birds) inside **sheds?** | Yes |  | Yes | |  | | Yes | |  | Yes | |  | Yes | |  |
|  | No |  | No | |  | | No | |  | No | |  | No | |  |
|  |  |  |  |  |  | |  |  |  |  |  |  |  |  |  |
|  | | | | | | | | | | | | | | | |
| 1. How often/ when do they visit the **sheds?** |  | | |  | | | |  | | |  | | |  | |
|  |  | |  | | | |  | |  | | | |  | | |
| 1. Have you observed any other wild creatures (besides birds) on the **range?** | Yes |  | Yes | | |  | Yes | |  | Yes | |  | Yes | |  |
|  | No |  | No | | |  | No | |  | No | |  | No | |  |
|  |  |  |  |  |  |  |  |  |  |  |  |  |  |  |  |
|  |  | |  | | | |  | |  | | | |  | | |
| 1. How often/ when do they visit the **range?** |  | | |  | | | |  | | |  | | |  | |
|  | | | | | | | | | | | | | | | |
| 1. Have you observed any other wild creatures (besides birds) in **feed storage areas?** | Yes |  | Yes | | |  | Yes | |  | Yes | |  | Yes | |  |
|  | No |  | No | | |  | No | |  | No | |  | No | |  |
|  |  |  |  |  |  |  |  |  |  |  |  |  |  |  |  |
|  |  | |  | | | |  | |  | | | |  | | |
| 1. How often/ when do they visit the **feed storage areas?** |  | | |  | | | |  | | |  | | |  | |
|  |  | |  | | | |  | |  | | | |  | | |
| 1. What types of interactions do they have with poultry? | Direct (physical) contact |  | Direct (physical) contact | |  | | Direct (physical) contact | |  | Direct (physical) contact | |  | Direct (physical) contact | |  |
|  |  |  |  |  |  | |  |  |  |  |  |  |  |  |  |
|  | Indirect contacting (perching in shed, eating feed, drinking water) |  | Indirect contacting (perching in shed, eating feed, drinking water) | |  | | Indirect contacting (perching in shed, eating feed, drinking water) | |  | Indirect contacting (perching in shed, eating feed, drinking water) | |  | Indirect contacting (perching in shed, eating feed, drinking water) | |  |
|  |  |  |  |  |  | |  |  |  |  |  |  |  |  |  |
|  |  |  |  |  |  | |  |  |  |  |  |  |  |  |  |
|  | None |  | None | |  | | None | |  | None | |  | None | |  |
|  |  | |  | | | |  | |  | | | |  | | |

| 1. What other animals are kept on the farm? | | | | | | | | |
| --- | --- | --- | --- | --- | --- | --- | --- | --- |
| Dogs, cats | | |  | | Other birds |  | Pigs |  |
| Ruminants | | |  | | Horses |  | None |  |
| Other | | |  | | | | | |
|  | | | | | | | | |
| 1. Do they have access to the insides of the chicken sheds or food storage areas? | | | | | | | | |
| Yes |  | No |  |  |  |  |  |  |
| Comments |  | | | | | | | |
|  | | | | | | | | |
| 1. If yes, how often do they visit these areas? | | | | | | | | |
|  | | | | | | | | |

**Farm movements**

**_____________________________________________________________________________________________**

| 1. What is the distance to the next farm and what type of farm is it? | | | | | | | | | | | | | | | | | | | | | | | | | | | | | | | | | | | | | | | | | | | | | | | | | | | | | |  |  |  |  |
| --- | --- | --- | --- | --- | --- | --- | --- | --- | --- | --- | --- | --- | --- | --- | --- | --- | --- | --- | --- | --- | --- | --- | --- | --- | --- | --- | --- | --- | --- | --- | --- | --- | --- | --- | --- | --- | --- | --- | --- | --- | --- | --- | --- | --- | --- | --- | --- | --- | --- | --- | --- | --- | --- | --- | --- | --- | --- |
|  | | | | | | | | | | | | | | | | | | | | | | | | | | | | | | | | | | | | | | | | | | | | | | | | | | | | | |  |  |  |  |
|  | | | | | | | | | | | | | | | | | | | | | | | | | | | | | | | | | | | | | | | | | | | | | | | | | | | | | |  |  |  |  |
| 1. What is the distance to the next poultry farm? | | | | | | | | | | | | | | | | | | | | | | | | | | | | | | | | | | | | | | | | | | | | | | | | | | | | | |  |  |  |  |
|  | | | | | | | | | | | | | | | | | | | | | | | | | | | | | | | | | | | | | | | | | | | | | | | | | | | | | |  |  |  |  |
|  | | | | | | | | | | | | | | | | | | | | | | | | | | | | | | | | | | | | | | | | | | | | | | | | | | | | | |  |  |  |  |
| 1. Is equipment (e.g. buckets, tools) on the farm dedicated to one shed only or is there sharing between sheds? | | | | | | | | | | | | | | | | | | | | | | | | | | | | | | | | | | | | | | | | | | | | | | | | | | | | | |  |  |  |  |
| One shed only | | |  | | | Sharing | | | | | |  | | | | Other | | | | | | | |  | | | | | | | | | | | | | | | | | | | | | | | | | | | | | |  |  |  |  |
|  | | | | | | | | | | | | | | | | | | | | | | | | | | | | | | | | | | | | | | | | | | | | | | | | | | | | | |  |  |  |  |
| 1. If shared, is equipment disinfected between sheds? | | | | | | | | | | | | | | | | | | | | | | | | | | | | | | | | | | | | | | | | | | | | | | | | | | | | | |  |  |  |  |
| Yes | | |  | | | No | | | | | |  | | | | Other | | | | | | | |  | | | | | | | | | | | | | | | | | | | | | | | | | | | | | |  |  |  |  |
|  | | | | | | | | | | | | | | | | | | | | | | | | | | | | | | | | | | | | | | | | | | | | | | | | | | | | | |  |  |  |  |
| 1. What method of shed sanitization is used? | | | | | | | | | | | | | | | | | | | | | | | | | | | | | | | | | | | | | | | | | | | | | | | | | | | | | |  |  |  |  |
| Removal of litter only | | | | | | | | | | |  | | Removal of litter and use of antiseptic/ disinfectant solution | | | | | | | | | | | | | | | | | | | | | | | | | | | | | | | | | | | |  | | | | |  |  |  |  |
| Other |  | | | | | | | | | | | | | | | | | | | | | | | | | | | | | | | | | | | | | | | | | | | | | | | | | | | | |  |  |  |  |
|  | | | | | | | | | | | | | | | | | | | | | | | | | | | | | | | | | | | | | | | | | | | | | | | | | | | | | |  |  |  |  |
| 1. How frequently are sheds sanitized? | | | | | | | | | | | | | | | | | | | | | | | | | | | | | | | | | | | | | | | | | | | | | | | | | | | | | |  |  |  |  |
| Between batches | | | | | | | |  | | | | | Once every half a year | | | | | | | | | | | | | | | | | | |  | | | Once a year | | | | | | | | | | | | | |  | | | | |  |  |  |  |
| Other | | | | | | | |  | | | | | | | | | | | | | | | | | | | | | | | | | | | | | | | | | | | | | | | | | | | | | |  |  |  |  |
|  | | | | | | | | | | | | | | | | | | | | | | | | | | | | | | | | | | | | | | | | | | | | | | | | | | | | | |  |  |  |  |
| 1. How are eggs collected? | | | | | | | | | | | | | | | | | | | | | | | | | | | | | | | | | | | | | | | | | | | | | | | | | | | | | |  |  |  |  |
| Manual collection | | | | |  | | Conveyor belt | | | | | | | | | | |  | | | | Other | | | | |  | | | | | | | | | | | | | | | | | | | | | | | | | |  |  |  |  |  |
|  | | | | | | | | | | | | | | | | | | | | | | | | | | | | | | | | | | | | | | | | | | | | | | | | | | | | | |  |  |  |  |
| 1. Where are the eggs graded (please specific the location)? | | | | | | | | | | | | | | | | | | | | | | | | | | | | | | | | | | | | | | | | | | | | | | | | | | | | | |  |  |  |  |
| On-site | | |  | | | Off-site | | | | | | | | |  | | | | | Location | | | | | | |  | | | | | | | | | | | | | | | | | | | | | | | | | |  |  |  |  |  |
|  | | | | | | | | | | | | | | | | | | | | | | | | | | | | | | | | | | | | | | | | | | | | | | | | | | | | | |  |  |  |  |
| 1. Where are eggs (please specify the location)? | | | | | | | | | | | | | | | | | | | | | | | | | | | | | | | | | | | | | | | | | | | | | | | | | | | | | |  |  |  |  |
| On-farm | | |  | | | Off-site | | | | | | | | |  | | | | | Location | | | | | | |  | | | | | | | | | | | | | | | | | | | | | | | | | |  |  |  |  |  |
|  | | | | | | | | | | | | | | | | | | | | | | | | | | | | | | | | | | | | | | | | | | | | | | | | | | | | |  |  |  |  |  |
| 1. Are egg trays sent or received by other farms? | | | | | | | | | | | | | | | | | | | | | | | | | | | | | 1. Are egg trays recycled? | | | | | | | | | | | | | | | | | | | | | | | |  |  |  |  |  |
| Yes | | |  | | | No | | | | | | | | |  | | | | |  | | | | | | | | | Yes | | | | | | | | |  | No | | | | | |  | | |  | | | | | | |  |  |  |
|  | | | | | | | | | | | | | | | | | | | | | | | | | | | | | | | | | | | | | | | | | | | | | | | | | | | | | |  |  |  |  |
|  | | | | | **Eggs** | | | | | | | | | | | | | | | | | **Day old chicks** | | | | | | | | | | **Reared birds** | | | | | | | | | | **Birds for slaughter** | | | | | | | | | | | | |  |  |  |
|  | | | | |  | | | | | | | | | | | | | | | | |  | | | | | | | | | |  | | | | | | | | | |  | | | | | | | | | | | | |  |  |  |
| 1. What material are the crates/boxes that the following are placed in for travel made out of? | | | | | Cardboard | | | | | | | | | | | | |  | | | | Cardboard | | | | | | | | |  | Cardboard | | | | | | | | |  | Cardboard | | | | | | | | | | |  | |  |  |  |
|  |  |  |  |  |  |  |  |  |  |  |  |  |  |  |  |  |  |  | | | |  |  |  |  |  |  |  |  |  |  |  |  |  |  |  |  |  |  |  |  |  |  |  |  |  |  |  |  |  |  |  |  | |  |  |  |
|  |  |  |  |  | Plastic | | | | | | | | | | | | |  | | | | Plastic | | | | | | | | |  | Plastic | | | | | | | | |  | Plastic | | | | | | | | | | |  | |  |  |  |
|  |  |  |  |  | Metal | | | | | | | | | | | | |  | | | | Metal | | | | | | | | |  | Metal | | | | | | | | |  | Metal | | | | | | | | | | |  | |  |  |  |
|  |  |  |  |  | Other | | | | |  | | | | | | | | | | | | Other | | | | |  | | | | | Other | | | | |  | | | | | Other | | | | |  | | | | | | | |  |  |  |
|  | | | | | | | | | | | | | | | | | | | | | | | | | | | | | | | | | | | | | | | | | | | | | | | | | | | | | | |  |  |  |
| 1. What company delivers/ takes away the birds? | | | | |  | | | | | |  | | | | | | | | | | |  | |  | | | | | | | |  | | |  | | | | | | |  | |  | | | | | | | | | | |  |  |  |
|  | | | | | | | | | | | | | | | | | | | | | | | | | | | | | | | | | | | | | | | | | | | | | | | | | | | | | | |  |  |  |
| 1. Have you seen them dirty on arrival? | | | | |  | | | | |  | | | | | | | | | | | |  | |  | | | | | | | |  | | |  | | | | | | |  | |  | | | | | | | | | | |  |  |  |
|  | | | | | | | | | | | | | | | | | | | | | | | | | | | | | | | | | | | | | | | | | | | | | | | | | | | | | | |  |  |  |
| 1. Which feed mill do you source feed from? | | | | | | | | | | | | | | | | | | | | | | | |  | | | | | | | | | | | | | | | | | | | | | | | | | | | | | | |  |  |  |
|  | | | | | | | | | | | | | | | | | | | | | | | | | | | | | | | | | | | | | | | | | | | | | | | | | | | | | | |  |  |  |
| 1. Which company delivers the feed? | | | | | | | | | | | | | | | | | | | | | | | |  | | | | | | | | | | | | | | | | | | | | | | | | | | | | | | |  |  |  |
|  | | | | | | | | | | | | | | | | | | | | | | | | | | | | | | | | | | | | | | | | | | | | | | | | | | | | | | |  |  |  |
| Comments | |  | | | | | | | | | | | | | | | | | | | | | | | | | | | | | | | | | | | | | | | | | | | | | | | | | | | |  |  |  |  |
|  | | | | | | | | | | | | | | | | | | | | | | | | | | | | | | | | | | | | |  | | | | |  | |  | | | | | | |  | | |  |  |  |  |
| 1. Do you send live birds to markets? | | | | | | | | | | | | | | | | | | | | | | | | | | | | | | | | | | | | | Yes | | | | |  | | No | | | | | | |  | | |  |  |  |  |
| Comments |  | | | | | | | | | | | | | | | | | | | | | | | | | | | | | | | | | | | | | | | | | | | | | | | | | | | | |  |  |  |  |
|  | | | | | | | | | | | | | | | | | | | | | | | | | | | | | | | | | | | | |  | | | | |  | |  | | | | | | |  | | |  |  |  |  |
| 1. Is there equipment or vehicle sharing between farms? | | | | | | | | | | | | | | | | | | | | | | | | | | | | | | | | | | | | | Yes | | | | |  | | No | | | | | | |  | | |  |  |  |  |
|  | | | | | | | | | | | | | | | | | | | | | | | | | | | | | | | | | | | | | | | | | | | | | | | | | | | | | |  |  |  |  |
| 1. If yes, what equipment and how often? | | | | | | | | | | | | | | | | | | | | | | | | | | | | | | | | | | | | | | | | | | | | | | | | | | | | | |  |  |  |  |
|  | | | | | | | | | | | | | | | | | | | | | | | | | | | | | | | | | | | | | | | | | | | | | | | | | | | | | |  |  |  |  |
|  | | | | | | | | | | | | | | | | | | | | | | | | | | | | | | | | | | | | |  | | | | |  | |  | | | | | | |  | | |  |  |  |  |
| 1. Does disinfection of equipment occur between farms? | | | | | | | | | | | | | | | | | | | | | | | | | | | | | | | | | | | | | Yes | | | | |  | | No | | | | | | |  | | |  |  |  |  |
|  | | | | | | | | | | | | | | | | | | | | | | | | | | | | | | | | | | | | | | | | | | | | | | | | | | | | | |  |  |  |  |
| 1. How are dead birds managed? | | | | | | | | | | | | | | | | | | | | | | | | | | | | | | | | | | | | | | | | | | | | | | | | | | | | | |  |  |  |  |
|  | | | | | | | | | | | | | | | | | | | | | | | | | | | | | | | | | | | | | | | | | | | | | | | | | | | | | |  |  |  |  |
| Composted on-site | | | | | | | | | |  | | | | Buried | | | | | | | | | | | | | | | | | | |  | | Incinerated | | | | | | | | | | | | | |  | | | | |  |  |  |  |
| Freezer | | | | | | | | | |  | | | | Other | | | | | | | | | | | | | | | | | | |  | | | | | | | | | | | | | | | | | | | | |  |  |  |  |
| Comments | | | | | | | | | |  | | | | | | | | | | | | | | | | | | | | | | | | | | | | | | | | | | | | | | | | | | | |  |  |  |  |
|  | | | | | | | | | | | | | | | | | | | | | | | | | | | | | | | | | | | | | | | | | | | | | | | | | | | | | |  |  |  |  |
| 1. How is chicken manure managed? | | | | | | | | | | | | | | | | | | | | | | | | | | | | | | | | | | | | | | | | | | | | | | | | | | | | | |  |  |  |  |
|  | | | | | | | | | | | | | | | | | | | | | | | | | | | | | | | | | | | | | | | | | | | | | | | | | | | | | |  |  |  |  |
| Composted on-site | | | | | | | |  | | | | | Stockpiled on farm | | | | | | | | | | | | | | | | | | |  | | | Given to off-site user | | | | | | | | | | | | | |  | | | | |  |  |  |  |
| Other | | | | | | | |  | | | | | | | | | | | | | | | | | | | | | | | | | | | | | | | | | | | | | | | | | | | | | |  |  |  |  |
| Comments | | | | | | | |  | | | | | | | | | | | | | | | | | | | | | | | | | | | | | | | | | | | | | | | | | | | | | |  |  |  |  |
|  | | | | | | | | | | | | | | | | | | | | | | | | | | | | | | | | | | | | | | | | | | | | | | | | | | | | | |  |  |  |  |
| 1. Are the following jobs performed by on-site farm staff or off-site people (circle appropriate)? Do they visit other farms? | | | | | | | | | | | | | | | | | | | | | | | | | | | | | | | | | | | | | | | | | | | | | | | | | | | | | |  |  |  |  |
|  | | | | | | | | | | | | | | | | | | | | | | | | | | | | | | | | | | | | | | | | | | | | | | | | | | | | | |  |  |  |  |
| Visit other farms | | | | | | | | | | | | | | | | | | | | | | | | | | | | Visit other farms | | | | | | | | | | | | | | | | | | | | | | | | | |  |  |  |  |
| Yes No | | | | | | | | | | | | | | | | | | | | | | | | | | | | Yes No | | | | | | | | | | | | | | | | | | | | | | | | | |  |  |  |  |
| Egg collectors *Off-site ● On-site* | | | | | | | | | | | | | | | | | | |  | | | | | |  | | | Meat bird collectors *Off-site ● On-site* | | | | | | | | | | | | | | | | | | |  | | |  | | | |  |  |  |  |
| Old litter removers *Off-site ● On-site* | | | | | | | | | | | | | | | | | | |  | | | | | |  | | | Fresh litter deliverers *Off-site ● On-site* | | | | | | | | | | | | | | | | | | |  | | |  | | | |  |  |  |  |
| Manure collectors *Off-site ● On-site* | | | | | | | | | | | | | | | | | | |  | | | | | |  | | | New bird deliverers *Off-site ● On-site* | | | | | | | | | | | | | | | | | | |  | | |  | | | |  |  |  |  |
| Shed sanitization people *Off-site ● On-site* | | | | | | | | | | | | | | | | | | |  | | | | | |  | | | Dead bird collectors *Off-site ● On-site* | | | | | | | | | | | | | | | | | | |  | | |  | | | |  |  |  |  |
| Electrician *Off-site ● On-site* | | | | | | | | | | | | | | | | | | |  | | | | | |  | | | Shed fixers *Off-site ● On-site* | | | | | | | | | | | | | | | | | | |  | | |  | | | |  |  |  |  |
| Plumber *Off-site ● On-site* | | | | | | | | | | | | | | | | | | |  | | | | | |  | | | Mechanic *Off-site ● On-site* | | | | | | | | | | | | | | | | | | |  | | |  | | | |  |  |  |  |
| Other *Off-site ● On-site* | | | | | | | | | | | | | | | | | | |  | | | | | |  | | |  | | | | | | | | | | | | | | | | | | |  | | |  | | | |  |  |  |  |
|  | | | | | | | | | | | | | | | | | | |  | | | | | |  | | |  | | | | | | | | | | | | | | | | | | |  | | |  | | | |  |  |  |  |
|  | | | | | | | | | | | | | | | | | | | | | | | | | | | | | | | | | | | | | | | | | | | | | | | | | | | | | |  |  |  |  |
