# Supplementary material for: Comparisons of management practices and farm design on Australian commercial layer and meat chicken farms: Cage, barn and free range
Source: PLoS One. 2017 Nov 22;12(11):e0188505. doi: 10.1371/journal.pone.0188505 (PMC5699831; doi:10.1371/journal.pone.0188505)
Supplement: S2 Text — (DOCX) [file pone.0188505.s002.docx]

**BIOSECURITY**

**_____________________________________________________________________________________________**

| 1. What does the term ‘biosecurity’ mean to you? | | | | | | | | | | | | | | | | | | | | | | | | | | | | | | | | | | | | | | | | | | |  |  |  |  |  |  |  |  |  |  |  |
| --- | --- | --- | --- | --- | --- | --- | --- | --- | --- | --- | --- | --- | --- | --- | --- | --- | --- | --- | --- | --- | --- | --- | --- | --- | --- | --- | --- | --- | --- | --- | --- | --- | --- | --- | --- | --- | --- | --- | --- | --- | --- | --- | --- | --- | --- | --- | --- | --- | --- | --- | --- | --- | --- |
|  | | | | | | | | | | | | | | | | | | | | | | | | | | | | | | | | | | | | | | | | | | |  |  |  |  |  |  |  |  |  |  |  |
| 1. Please tick **who** you obtain information regarding poultry health, diseases and news. Please also rate how reliable you believe **ALL** of the following sources are. | | | | | | | | | | | | | | | | | | | | | | | | | | | | | | | | | | | | | | | | | | |  |  |  |  |  |  |  |  |  |  |  |
|  | | | | | | | ✓ | | | Not reliable | | | | | | | Slightly reliable | | | | | | | | | | | | | | | | | Moderately reliable | | | | | Very reliable | | | Unsure |  |  |  |  |  |  |  |  |  |  |  |
| Other poultry farmers/ friends | | | | | | |  | | | O | | | | | | | O | | | | | | | | | | | | | | | | | O | | | | | O | | | O |  |  |  |  |  |  |  |  |  |  |  |
|  | | | | | | | | | | | | | | | | | | | | | | | | | | | | | | | | | | | | | | | | | | |  | |  | |  | |  | |  |  |  |
| Poultry organizations (e.g. Australian Egg Corporation Limited, Australian Chicken Meat Federation) | | | | | | | |  | | | | O | | | | O | | | | | | | | | | | | | | | | | O | | | | | | O | | | O |  |  |  |  |  |  |  |  |  |  |  |
|  |  |  |  |  |  |  |  |  | | | |  |  |  |  |  |  |  |  |  |  |  |  |  |  |  |  |  |  |  |  |  |  |  |  |  |  |  |  |  |  |  |  |  |  |  |  |  |  |  |  |  |  |
| Please specify the poultry organization(s) | | | | | | | | | | | | | | | | | | | | | | | | | | | | | | | | | | | | | | | | | | |  | |  | |  | |  | |  |  |  |
|  | |  | | | | | | | | | | | | | | | | | | | | | | | | | | | | | | | | | | | | | | | | |  |  |  |  |  |  |  |  |  |  |  |
|  | | | | | | | | | | | | | | | | | | | | | | | | | | | | | | | | | | | | | | | | | | |  |  |  |  |  |  |  |  |  |  |  |
| Integrators (e.g. Pace, Baiada) | | | | | | | |  | | | | O | | | O | | | | | | | | | | | | | | | | | O | | | | | | O | | | | O |  |  |  |  |  |  |  |  |  |  |  |
| Please specify the integrator(s) | | | | | | | | | | | | | | | | | | | | | | | | | | | | | | | | | | | | | | | | | | |  | |  | |  | |  | |  |  |  |
|  | |  | | | | | | | | | | | | | | | | | | | | | | | | | | | | | | | | | | | | | | | | |  |  |  |  |  |  |  |  |  |  |  |
|  | |  | | | | | | | | | | | | | | | | | | | | | | | | | | | | | | | | | | | | | | | | |  |  |  |  |  |  |  |  |  |  |  |
| Veterinarian | |  | | | | | |  | | | | O | | | | | O | | | | | | | | | | | | | | | | | O | | | | | O | | | O |  |  |  |  |  |  |  |  |  |  |  |
|  | |  | | | | | | | | | | | | | | | | | | | | | | | | | | | | | | | | | | | | | | | | |  | |  | |  | |  | |  |  |  |
| Independent consultants | |  | | | | | |  | | | | O | | | | | O | | | | | | | | | | | | | | | | | O | | | | | O | | | O |  |  |  |  |  |  |  |  |  |  |  |
|  | | | | | | | | | | | | | | | | | | | | | | | | | | | | | | | | | | | | | | | | | | |  | |  | |  | |  | |  |  |  |
| Other (please specify) | |  | | | | | |  | | | | | O | | | O | | | | | | | | | | | | | | | | | O | | | | | | O | | | O |  |  |  |  |  |  |  |  |  |  |  |
|  | | | | | | | | | | | | |  | | | | | | | | | | | | | | | | | | | | | | | | | | | | | |  | |  | |  | |  | |  |  |  |
|  | |  | | | | | | | | | | | | | | | | | | | | | | | | | | | | | | | | | | | | | | | | |  |  |  |  |  |  |  |  |  |  |  |
| Any further comments | |  | | | | | | | | | | | | | | | | | | | | | | | | | | | | | | | | | | | | | | | | |  |  |  |  |  |  |  |  |  |  |  |
|  | | | | | | | | | | | | | | | | | | | | | | | | | | | | | | | | | | | | | | | | | | |  |  |  |  |  |  |  |  |  |  |  |
| 1. Please tick **how** you obtain information regarding poultry health, diseases and news. Please also rate how reliable you believe **ALL** of the following methods of information delivery are. | | | | | | | | | | | | | | | | | | | | | | | | | | | | | | | | | | | | | | | | | | |  |  |  |  |  |  |  |  |  |  |  |
|  | | | | | | | | | | | | | | | | | | | | | | | | | | | | | | | | | | | | | | | | | | |  |  |  |  |  |  |  |  |  |  |  |
|  | | | | | | | | | ✓ | | | | Not reliable | | | | | Slightly reliable | | | | | | | | | | | | | | | | | Moderately reliable | | | | | Very reliable | | Unsure |  |  |  |  |  |  |  |  |  |  |  |
| Social media websites (e.g. Twitter, Facebook) | | | | | | | | |  | | | | O | | | | | O | | | | | | | | | | | | | | | | | O | | | | | O | | O |  |  |  |  |  |  |  |  |  |  |  |
| Please specify the website(s) | | | | | | | | | | | | | | | | | | | | | | | | | | | | | | | | | | | | | | | | | | |  |  |  |  |  |  |  |  |  |  |  |
|  | | |  | | | | | | | | | | | | | | | | | | | | | | | | | | | | | | | | | | | | | | | |  |  |  |  |  |  |  |  |  |  |  |
|  | | | | | | | | | | | | | | | | | | | | | | | | | | | | | | | | | | | | | | | | | | |  |  |  |  |  |  |  |  |  |  |  |
| Subscribed emails | | | | | | | | |  | | | | O | | | | | O | | | | | | | | | | | | | | | | | O | | | | | O | | O |  |  |  |  |  |  |  |  |  |  |  |
| Please specify who the email(s) is/are from | | | | | | | | | | | | | | | | | | | | | | | | | | | | | | | | | | | | | | | | | | |  |  |  |  |  |  |  |  |  |  |  |
|  | | |  | | | | | | | | | | | | | | | | | | | | | | | | | | | | | | | | | | | | | | | |  |  |  |  |  |  |  |  |  |  |  |
|  | | | | | | | | | | | | | | | | | | | | | | | | | | | | | | | | | | | | | | | | | | |  |  |  |  |  |  |  |  |  |  |  |
| Official websites (e.g. government websites, company websites) | | | | | | | | |  | | | | O | | | | | | O | | | | | | | | | | | | | | | | O | | | | | O | | O |  |  |  |  |  |  |  |  |  |  |  |
|  |  |  |  |  |  |  |  |  |  | | | |  |  |  |  |  |  |  |  |  |  |  |  |  |  |  |  |  |  |  |  |  |  |  |  |  |  |  |  |  |  |  |  |  |  |  |  |  |  |  |  |  |
| Please specify the website(s) | | | | | | | | | | | | | | | | | | | | | | | | | | | | | | | | | | | | | | | | | | |  |  |  |  |  |  |  |  |  |  |  |
|  | | |  | | | | | | | | | | | | | | | | | | | | | | | | | | | | | | | | | | | | | | | |  |  |  |  |  |  |  |  |  |  |  |
|  | | | | | | | | | | | | | | | | | | | | | | | | | | | | | | | | | | | | | | | | | | |  |  |  |  |  |  |  |  |  |  |  |
| Newsletter (paper-based or electronic) | | | | | | | | |  | | | | O | | | | | | | O | | | | | | | | | | | | | | | | O | | | | O | | O |  |  |  |  |  |  |  |  |  |  |  |
| Please specify who the newsletter(s) is/are from | | | | | | | | | | | | | | | | | | | | | | | | | | | | | | | | | | | | | | | | | | |  |  |  |  |  |  |  |  |  |  |  |
|  | | |  | | | | | | | | | | | | | | | | | | | | | | | | | | | | | | | | | | | | | | | |  |  |  |  |  |  |  |  |  |  |  |
|  | | | | | | | | | | | | | | | | | | | | | | | | | | | | | | | | | | | | | | | | | | |  |  |  |  |  |  |  |  |  |  |  |
| Television/radio | | | | | | | | |  | | | | O | | | | | | | O | | | | | | | | | | | | | | | | O | | | | O | | O |  |  |  |  |  |  |  |  |  |  |  |
| Please specify the station/program(s) | | | | | | | | | | | | | | | | | | | | | | | | | | | | | | | | | | | | | | | | | | |  |  |  |  |  |  |  |  |  |  |  |
|  | | |  | | | | | | | | | | | | | | | | | | | | | | | | | | | | | | | | | | | | | | | |  |  |  |  |  |  |  |  |  |  |  |
|  | | |  | | | | | | | | | | | | | | | | | | | | | | | | | | | | | | | | | | | | | | | |  |  |  |  |  |  |  |  |  |  |  |
| Other (please specify) | | | | | | | | | | | | | | | | | | | | | | | | | | | | | | | | | | | | | | | | | | |  |  |  |  |  |  |  |  |  |  |  |
|  | | | | | | | | | | | | |  | | | | | | | | | | | | | | | | | | | | | | | | | | | | | |  |  |  |  |  |  |  |  |  |  |  |
|  | | | | | | | | | | | | |  | | | | | | | | | | | | | | | | | | | | | | | | | | | | | |  |  |  |  |  |  |  |  |  |  |  |
| Any further comments | | |  | | | | | | | | | | | | | | | | | | | | | | | | | | | | | | | | | | | | | | | |  |  |  |  |  |  |  |  |  |  |  |
|  | | | | | | | | | | | | | | | | | | | | | | | | | | | | | | | | | | | | | | | | | | |  |  |  |  |  |  |  |  |  |  |  |
| 1. For the following activities, please tick which ones are performed on this farm. Please also rate **ALL** of the following activities from 1 to 5 which, in your opinion, are important in the prevention, introduction and spread of diseases (1 being unimportant, 5 being very important). | | | | | | | | | | | | | | | | | | | | | | | | | | | | | | | | | | | | | | | | | | |  |  |  |  |  |  |  |  |  |  |  |
|  | | | | | | | | | | | | | | | | | | | | | | | | | | | | | | | | | | | | | | | | | | |  |  |  |  |  |  |  |  |  |  |  |
|  | | | |  | | Unimportant | | | | | | | | Slightly important | | | | | | | Moderately important | | | | | | | | | | | | | | | | Very important | | | | Extremely important | |  |  |  |  |  |  |  |  |  |  |  |
|  | | | |  | |  | | | | | | | | | | | | | | | | | | | | | | | | | | | | | | | | | | |  | |  |  |  |  |  |  |  |  |  |  |  |
| **General** | | | | ✓ | |  | | | | | | | | | | | | | | | | | | | | | | | | | | | | | | | | | | |  | |  |  |  |  |  |  |  |  |  |  |  |
|  | | | |  | |  | | | | | | | | | | | | | | | | | | | | | | | | | | | | | | | | | | |  | |  |  |  |  |  |  |  |  |  |  |  |
| Industry-based biosecurity plan/manual on farm | | | |  | | O | | | | | O | | | | | | | | | | O | | | | | | | | | | | | | | | | O | | | | O | |  |  |  |  |  |  |  |  |  |  |  |
|  |  |  |  |  | |  |  |  |  |  |  |  |  |  |  |  |  |  |  |  |  |  |  |  |  |  |  |  |  |  |  |  |  |  |  |  |  |  |  |  |  |  |  |  |  |  |  |  |  |  |  |  |  |
|  | | | |  | |  | | | | |  | | | | | | | | | |  | | | | | | | | | | | | | | | |  | | | |  | |  |  |  |  |  |  |  |  |  |  |  |
| Fence around farm | | | |  | | O | | | | | O | | | | | | | | | | | O | | | | | | | | | | | | | | | O | | | | O | |  |  |  |  |  |  |  |  |  |  |  |
|  |  |  |  |  | |  |  |  |  |  |  |  |  |  |  |  |  |  |  |  |  |  |  |  |  |  |  |  |  |  |  |  |  |  |  |  |  |  |  |  |  |  |  |  |  |  |  |  |  |  |  |  |  |
| **For both workers and visitors** | | | | | | | | | | | | | | | | | | | | | | | | | | | | | | | | | | | | | | | | | | |  |  |  |  |  |  |  |  |  |  |  |
|  | | | |  | |  | | | | | | | | | | | | | | | | | | | | | | | | | | | | | | | | | | |  | |  |  |  |  |  |  |  |  |  |  |  |
| Compulsory visitor recording system (e.g. log book) | | | |  | | O | | | | | O | | | | | | | | | | O | | | | | | | | | | | | | | | | O | | | | O | |  |  |  |  |  |  |  |  |  |  |  |
|  |  |  |  |  | |  |  |  |  |  |  |  |  |  |  |  |  |  |  |  |  |  |  |  |  |  |  |  |  |  |  |  |  |  |  |  |  |  |  |  |  |  |  |  |  |  |  |  |  |  |  |  |  |
|  | | | |  | |  | | | | | | | | | | | | | | | | | | | | | | | | | | | | | | | | | | |  | |  |  |  |  |  |  |  |  |  |  |  |
| Written instructions for visitors at the entrance of the farm | | | |  | | O | | | | | O | | | | | | | | | | | O | | | | | | | | | | | | | | | O | | | | O | |  |  |  |  |  |  |  |  |  |  |  |
|  |  |  |  |  | |  |  |  |  |  |  |  |  |  |  |  |  |  |  |  |  |  |  |  |  |  |  |  |  |  |  |  |  |  |  |  |  |  |  |  |  |  |  |  |  |  |  |  |  |  |  |  |  |
|  | | | |  | |  | | | | | | | | | | | | | | | | | | | | | | | | | | | | | | | | | | |  | |  |  |  |  |  |  |  |  |  |  |  |
| Visitors park their vehicle outside the farm/ designated parking space | | | |  | | O | | | | | O | | | | | | | | | | | O | | | | | | | | | | | | | | | O | | | | O | |  |  |  |  |  |  |  |  |  |  |  |
|  |  |  |  |  | |  |  |  |  |  |  |  |  |  |  |  |  |  |  |  |  |  |  |  |  |  |  |  |  |  |  |  |  |  |  |  |  |  |  |  |  |  |  |  |  |  |  |  |  |  |  |  |  |
|  |  |  |  |  | |  |  |  |  |  |  |  |  |  |  |  |  |  |  |  |  |  |  |  |  |  |  |  |  |  |  |  |  |  |  |  |  |  |  |  |  |  |  |  |  |  |  |  |  |  |  |  |  |
| Disinfection of vehicles onto and out of farm | | | |  | | O | | | | | O | | | | | | | | | | | O | | | | | | | | | | | | | | | O | | | | O | |  |  |  |  |  |  |  |  |  |  |  |
|  |  |  |  |  | |  |  |  |  |  |  |  |  |  |  |  |  |  |  |  |  |  |  |  |  |  |  |  |  |  |  |  |  |  |  |  |  |  |  |  |  |  |  |  |  |  |  |  |  |  |  |  |  |
|  | | | |  | |  | | | | |  | | | | | | | | | | |  | | | | | | | | | | | | | | |  | | | |  | |  |  |  |  |  |  |  |  |  |  |  |
| Disinfection of equipment between farms | | | |  | | O | | | | | O | | | | | | | | | | | O | | | | | | | | | | | | | | | O | | | | O | |  |  |  |  |  |  |  |  |  |  |  |
|  |  |  |  |  | |  |  |  |  |  |  |  |  |  |  |  |  |  |  |  |  |  |  |  |  |  |  |  |  |  |  |  |  |  |  |  |  |  |  |  |  |  |  |  |  |  |  |  |  |  |  |  |  |
|  | | | |  | |  | | | | |  | | | | | | | | | | |  | | | | | | | | | | | | | | |  | | | |  | |  |  |  |  |  |  |  |  |  |  |  |
| Shower in shower out facilities on farm | | | |  | | O | | | | | O | | | | | | | | | | | O | | | | | | | | | | | | | | | O | | | | O | |  |  |  |  |  |  |  |  |  |  |  |
|  |  |  |  |  | |  |  |  |  |  |  |  |  |  |  |  |  |  |  |  |  |  |  |  |  |  |  |  |  |  |  |  |  |  |  |  |  |  |  |  |  |  |  |  |  |  |  |  |  |  |  |  |  |
|  | | | |  | |  | | | | |  | | | | | | | | | | |  | | | | | | | | | | | | | | |  | | | |  | |  |  |  |  |  |  |  |  |  |  |  |
| Use of clothes and boots provided by the farm | | | |  | | O | | | | | O | | | | | | | | | | | O | | | | | | | | | | | | | | | O | | | | O | |  |  |  |  |  |  |  |  |  |  |  |
|  |  |  |  |  | |  |  |  |  |  |  |  |  |  |  |  |  |  |  |  |  |  |  |  |  |  |  |  |  |  |  |  |  |  |  |  |  |  |  |  |  |  |  |  |  |  |  |  |  |  |  |  |  |
|  | | | |  | |  | | | | | | | | | | | | | | | | | | | | | | | | | | | | | | | | | | |  | |  |  |  |  |  |  |  |  |  |  |  |
| Changing protective clothing/footwear between farms | | | |  | | O | | | | | O | | | | | | | | | | | | O | | | | | | | | | | | | | | O | | | | O | |  |  |  |  |  |  |  |  |  |  |  |
|  |  |  |  |  | |  |  |  |  |  |  |  |  |  |  |  |  |  |  |  |  |  |  |  |  |  |  |  |  |  |  |  |  |  |  |  |  |  |  |  |  |  |  |  |  |  |  |  |  |  |  |  |  |
|  | | | |  | |  | | | | | | | | | | | | | | | | | | | | | | | | | | | | | | | | | | |  | |  |  |  |  |  |  |  |  |  |  |  |
| Changing protective clothing/footwear between sheds | | | |  | | O | | | | | O | | | | | | | | | | | | O | | | | | | | | | | | | | | O | | | | O | |  |  |  |  |  |  |  |  |  |  |  |
|  |  |  |  |  | |  |  |  |  |  |  |  |  |  |  |  |  |  |  |  |  |  |  |  |  |  |  |  |  |  |  |  |  |  |  |  |  |  |  |  |  |  |  |  |  |  |  |  |  |  |  |  |  |
|  | | | |  | |  | | | | | | | | | | | | | | | | | | | | | | | | | | | | | | | | | | |  | |  |  |  |  |  |  |  |  |  |  |  |
| Workers do not have contact with other poultry farms/birds (including other farms with the same owner) | | | |  | | O | | | | | O | | | | | | | | | | | | O | | | | | | | | | | | | | | O | | | | O | |  |  |  |  |  |  |  |  |  |  |  |
|  |  |  |  |  | |  |  |  |  |  |  |  |  |  |  |  |  |  |  |  |  |  |  |  |  |  |  |  |  |  |  |  |  |  |  |  |  |  |  |  |  | |  |  |  |  |  |  |  |  |  |  |  |
|  | | | |  | |  | | | | | | | | | | | | | | | | | | | | | | | | | | | | | | | | | | |  | |  |  |  |  |  |  |  |  |  |  |  |
| **For sheds** | | | |  | |  | | | | | | | | | | | | | | | | | | | | | | | | | | | | | | | | | | |  | |  |  |  |  |  |  |  |  |  |  |  |
|  | | | |  | |  | | | | | | | | | | | | | | | | | | | | | | | | | | | | | | | | | | |  | |  |  |  |  |  |  |  |  |  |  |  |
| Footbaths in and out of sheds | | | |  | | O | | | | | O | | | | | | | | | | | | | O | | | | | | | | | | | | | O | | | | O | |  |  |  |  |  |  |  |  |  |  |  |
|  | | | |  | |  | | | | |  | | | | | | | | | | | | |  | | | | | | | | | | | | |  | | | |  | |  |  |  |  |  |  |  |  |  |  |  |
|  | | | |  | | Unimportant | | | | | Slightly important | | | | | | | | | | | | | Moderately important | | | | | | | | | | | | | Very important | | | | Extremely important | |  |  |  |  |  |  |  |  |  |  |  |
|  | | | |  | |  | | | | |  | | | | | | | | | | | | |  | | | | | | | | | | | | |  | | | |  | |  |  |  |  |  |  |  |  |  |  |  |
| Disinfect vehicles in between sheds | | | |  | | O | | | | | O | | | | | | | | | | | O | | | | | | | | | | | | | | | O | | | | O | |  |  |  |  |  |  |  |  |  |  |  |
|  |  |  |  |  | |  |  |  |  |  |  |  |  |  |  |  |  |  |  |  |  |  |  |  |  |  |  |  |  |  |  |  |  |  |  |  |  |  |  |  |  |  |  |  |  |  |  |  |  |  |  |  |  |
| Disinfection of equipment between sheds | | | |  | | O | | | | | O | | | | | | | | | | | | | O | | | | | | | | | | | | | O | | | | O | |  |  |  |  |  |  |  |  |  |  |  |
|  |  |  |  |  | |  |  |  |  |  |  |  |  |  |  |  |  |  |  |  |  |  |  |  |  |  |  |  |  |  |  |  |  |  |  |  |  |  |  |  |  |  |  |  |  |  |  |  |  |  |  |  |  |
|  | | | |  | |  | | | | | | | | | | | | | | | | | | | | | | | | | | | | | | | | | | |  | |  |  | |  | |  |  |  |  |  |  |
| Wild bird proofing sheds | | | |  | | O | | | | | O | | | | | | | | | | | | | | O | | | | | | | | | | | | O | | | | O | |  |  |  |  |  |  |  |  |  |  |  |
|  | | | |  | |  | | | | | | | | | | | | | | | | | | | | | | | | | | | | | | | | | | |  | |  |  | |  | |  |  |  |  |  |  |
| Wild bird proofing dams (e.g. orchard netting) | | | |  | | O | | | | | O | | | | | | | | | | | | | | O | | | | | | | | | | | | O | | | | O | |  |  |  |  |  |  |  |  |  |  |  |
|  |  |  |  |  | |  |  |  |  |  |  |  |  |  |  |  |  |  |  |  |  |  |  |  |  |  |  |  |  |  |  |  |  |  |  |  |  |  |  |  |  |  |  |  |  |  |  |  |  |  |  |  |  |
|  | | | |  | |  | | | | | | | | | | | | | | | | | | | | | | | | | | | | | | | | | | |  | |  |  |  |  |  |  |  |  |  |  |  |
| Rodent and other wild animal control in sheds | | | |  | | O | | | | | O | | | | | | | | | | | | | | | O | | | | | | | | | | | O | | | | O | |  |  |  |  |  |  |  |  |  |  |  |
|  |  |  |  |  | |  |  |  |  |  |  |  |  |  |  |  |  |  |  |  |  |  |  |  |  |  |  |  |  |  |  |  |  |  |  |  |  |  |  |  |  |  |  |  |  |  |  |  |  |  |  |  |  |
|  | | | |  | |  | | | | | | | | | | | | | | | | | | | | | | | | | | | | | | | | | | |  | |  |  |  |  |  |  |  |  |  |  |  |
| Insect/spider control in sheds | | | |  | | O | | | | | O | | | | | | | | | | | | | | | O | | | | | | | | | | | O | | | | O | |  |  |  |  |  |  |  |  |  |  |  |
|  | | | |  | |  | | | | | | | | | | | | | | | | | | | | | | | | | | | | | | | | | | |  | |  |  | |  | |  |  |  |  |  |  |
| Animals not allowed inside sheds (including pets and other livestock) | | | |  | | O | | | | | O | | | | | | | | | | | | | | | | O | | | | | | | | | | O | | | | O | |  |  |  |  |  |  |  |  |  |  |  |
|  |  |  |  |  | |  |  |  |  |  |  |  |  |  |  |  |  |  |  |  |  |  |  |  |  |  |  |  |  |  |  |  |  |  |  |  |  |  |  |  |  |  |  |  |  |  |  |  |  |  |  |  |  |
|  | | | |  | |  | | | | | | | | | | | | | | | | | | | | | | | | | | | | | | | | | | |  | |  |  |  |  |  |  |  |  |  |  |  |
| Hand washing facilities on farm and hand washing required before and after handling birds | | | |  | | O | | | | | O | | | | | | | | | | | | | | | | O | | | | | | | | | | O | | | | O | |  |  |  |  |  |  |  |  |  |  |  |
|  |  |  |  |  | |  |  |  |  |  |  |  |  |  |  |  |  |  |  |  |  |  |  |  |  |  |  |  |  |  |  |  |  |  |  |  |  |  |  |  |  | |  |  |  |  |  |  |  |  |  |  |  |
|  | | | |  | |  | | | | | | | | | | | | | | | | | | | | | | | | | | | | | | | | | | |  | |  |  |  |  |  |  |  |  |  |  |  |
| **Management of new stock and between batches** | | | |  | |  | | | | |  | | | | | | | | | | | | | | | | |  | | | | | | | | |  | | | |  | |  |  |  |  |  |  |  |  |  |  |  |
|  | | | |  | |  | | | | |  | | | | | | | | | | | | | | | | |  | | | | | | | | |  | | | |  | |  |  |  |  |  |  |  |  |  |  |  |
| Quarantine of new stock from existing birds on farm for at least 14 days before introduction on farm | | | |  | | O | | | | | O | | | | | | | | | | | | | | | | | O | | | | | | | | | O | | | | O | |  |  |  |  |  |  |  |  |  |  |  |
|  |  |  |  |  | |  |  |  |  |  |  |  |  |  |  |  |  |  |  |  |  |  |  |  |  |  |  |  |  |  |  |  |  |  |  |  |  |  |  |  |  | |  |  |  |  |  |  |  |  |  |  |  |
|  | | | |  | |  | | | | | | | | | | | | | | | | | | | | | | | | | | | | | | | | | | |  | |  |  |  |  |  |  |  |  |  |  |  |
| Knowledge of source of new stock | | | |  | | O | | | | | O | | | | | | | | | | | | | | | | | O | | | | | | | | | O | | | | O | |  |  |  |  |  |  |  |  |  |  |  |
|  | | | |  | |  | | | | | | | | | | | | | | | | | | | | | | | | | | | | | | | | | | |  | |  |  | |  | |  |  |  |  |  |  |
| Manure removal for each batch | | | |  | | O | | | | | O | | | | | | | | | | | | | | | | | | O | | | | | | | | O | | | | O | |  |  |  |  |  |  |  |  |  |  |  |
|  | | | |  | |  | | | | | | | | | | | | | | | | | | | | | | | | | | | | | | | | | | |  | |  |  | |  | |  |  |  |  |  |  |
| Fresh litter/shed sanitization for each new batch | | | |  | | O | | | | | O | | | | | | | | | | | | | | | | | | O | | | | | | | | O | | | | O | |  |  |  |  |  |  |  |  |  |  |  |
|  |  |  |  |  | |  |  |  |  |  |  |  |  |  |  |  |  |  |  |  |  |  |  |  |  |  |  |  |  |  |  |  |  |  |  |  |  |  |  |  |  |  |  |  |  |  |  |  |  |  |  |  |  |
|  | | | |  | |  | | | | | | | | | | | | | | | | | | | | | | | | | | | | | | | | | | |  | |  |  |  |  |  |  |  |  |  |  |  |
| Waiting an adequate length of time shed is empty (turnaround time) before introduction of new stock to shed | | | |  | | O | | | | | O | | | | | | | | | | | | | | | | | | | O | | | | | | | O | | | | O | |  |  |  |  |  |  |  |  |  |  |  |
|  |  |  |  |  | |  |  |  |  |  |  |  |  |  |  |  |  |  |  |  |  |  |  |  |  |  |  |  |  |  |  |  |  |  |  |  |  |  |  |  |  | |  |  |  |  |  |  |  |  |  |  |  |
|  |  | | | | | | | | | | | | | | | | | | | | | | | | | | | | | | | | | | | | | | | |  | |  |  | |  | |  | |  |  |  |  |
| Other (please specify) |  | | | | O | | | | | O | | | | | | | | | | | | | | | | | | | | | O | | | | | | O | | | | O | |  |  |  |  |  |  |  |  |  |  |  |
|  | | | | |  |  |  |  |  |  |  |  |  |  |  |  |  |  |  |  |  |  |  |  |  |  |  |  |  |  |  |  |  |  |  |  |  |  |  |  |  |  |  |  |  |  |  |  |  |  |  |  |  |

|  | | | | |
| --- | --- | --- | --- | --- |
| 1. What biosecurity rating would you give your farm? | | | | |
|  | | | | |
| You do not believe you follow biosecurity measures at all | You believe you follow biosecurity measures minimally | You believe you follow biosecurity measures occasionally | You believe you follow biosecurity measures most of the time | You believe you accomplish all biosecurity measures thoroughly |
| O | O | O | O | O |
|  | | | | |
| 1. How likely do you believe an avian influenza outbreak will occur on your farm? | | | | |
|  | | | | |
| Not at all likely | Unlikely | Likely | Very likely | Unsure |
| O | O | O | O | O |
|  | | | | |
| 1. How likely do you believe an avian influenza outbreak will occur on another farm in Australia in the next year? | | | | |
|  | | | | |
| Not at all likely | Unlikely | Likely | Very likely | Unsure |
| O | O | O | O | O |
